# Carbon dioxide emissions through land use change, fire, and oxidative peat decomposition in Borneo

**DOI:** 10.1038/s41598-023-40333-z

**Published:** 2023-08-11

**Authors:** Tomohiro Shiraishi, Ryuichi Hirata, Masato Hayashi, Takashi Hirano

**Affiliations:** 1 Earth System Division, National Institute for Environmental Studies (NIES), Ibaraki, 305-8506 Japan; 2 School of Engineering, Nippon Bunri University, Oita, 870-0397 Japan; 3 Earth Observation Research Center, Japan Aerospace Exploration Agency (JAXA), Ibaraki, 305-8505 Japan; 4 Research Faculty of Agriculture, Hokkaido University, Hokkaido, 060-8589 Japan

**Keywords:** Environmental impact, Climate-change impacts, Environmental impact

## Abstract

Borneo has accumulated an abundance of woody carbon in its forests and peat. However, agricultural land conversion accompanied by plantation development, dead wood burning, and peat drying from drainage are major challenges to climate change mitigation. This study aimed to develop a method of estimating carbon dioxide (CO_2_) emissions from land use change, forest and peat fires, and oxidative peat decomposition, and CO_2_ uptake from biomass growth across Borneo using remote sensing data from 2001 to 2016. Although CO_2_ uptake by biomass growth in vast forests has shown a significant increasing trend, an annual net release of 461.10 ± 436.51 (average ± 1 standard deviation) Tg CO_2_ year^−1^ was observed. The estimated emissions were predominantly characterized by land use changes from 2001 to 2003, with the highest emissions in 2001. Land use change was evaluated from annual land use maps with an accuracy of 92.0 ± 1.0% (average ± 1 standard deviation). Forest and peat fires contributed higher emissions in 2002, 2006, 2009, 2014, and 2015 compared to other years and were strongly correlated with the Southern Oscillation Indexes. These results suggest that more CO_2_ may have been released into the atmosphere than previously thought.

## Introduction

Forests play a vital role in the global carbon cycle by absorbing atmospheric carbon dioxide (CO_2_) and storing it as part of tree biomass. A total of 230 Mha of forest was lost through logging and land use change and 80 Mha of new forests were gained worldwide between 2000 and 2012^1^. Global net deforestation rates have steadily decreased from 7.8 Mha year^−1^ in 1990 − 2000, 5.2 Mha year^−1^ in 2000 − 2010, and 4.7 Mha year^−1^ in 2010 − 2020^2^. Currently, a total global forest area of 4.06 billion hectares remains, accounting for 31% of the total land area. Of this, the total spatial area of intact natural forest is only 27%^2^. The average for terrestrial CO_2_ sinks from 2010 to 2021 was estimated at 3.2 Pg C year^−1^, whereas land use changes, predominantly from deforestation, emitted 1.3 Pg C year^−1^ based on the Global Carbon Budget 2022^3^. Total net greenhouse gas (GHG) emissions from agriculture, forestry, and other land use (AFOLU) sectors were 12.0 Pg CO_2_ year^−1^ (CO_2_ equivalent) from 2007 to 2016^4^, accounting for 23% of total net anthropogenic emissions. Therefore, GHG emissions from AFOLU sectors have contributed substantially to the rise in atmospheric GHG concentrations.

The deforestation rate in Southeast Asia is high, especially in Indonesia, which saw the highest rate of forest loss from 2000 to 2012^1,5^. Although forests covered 71% of the land area of Borneo in the 1980s, this ratio decreased to 54% in 2000^6^ and decreased further by 14% (6.04 Mha) between 2000 and 2017^7^. In contrast, the total plantation area, such as for oil palm and pulpwood, expanded by 170% (6.20 Mha) in 2000–2017^7^. The main causes of deforestation in Borneo include the expansion of agricultural activity, conversion of forests to oil palm and pulpwood plantations, and logging^8,9^. Agricultural development has played a key role in contributing to regional economic growth in many tropical countries because of the low costs of labour and land^10^; however, this has also caused the extensive loss of natural habitats for native biodiversity, reduction in woody biomass, deterioration in water quality from drainage channels, increased GHG release from drying soil in peatlands, and financial losses and physical damage from the haze caused by biomass and peat burning^11^.

A large proportion of carbon is stored as peat. In insular Southeast Asia, a considerable amount of incompletely decomposed woody carbon has accumulated as peat under swamp forests over thousands of years^12^. The carbon content of peat is estimated at approximately 68.5 Pg, corresponding to 77% of global tropical peat carbon pool^12^. Land use change has the potential to transform the carbon pool and emit substantial amounts of CO_2_ into the atmosphere^12,13^. Peatlands in Southeast Asia have emitted a vast amount of CO_2_ through deforestation from land use changes^9,13^, drainage^14,15^, and fires^16,17^ over the last four decades. Southeast Asia is also sensitive to El Niño events^18^. In Borneo, the El Niño Southern Oscillation (ENSO) causes droughts by delaying the beginning of the rainy season^19–21^, thereby increasing the risk of forest fires^21^, accelerating oxidative peat decomposition, and, consequently, increasing CO_2_ emissions^22,23^.

To assess the carbon balance of tropical ecosystems under human pressure, it is essential to quantify CO_2_ emissions through land use changes accompanied by deforestation and drainage, and forest and peat fires. However, the precise assessment of CO_2_ emissions from peatland ecosystems remains difficult because of a lack of field data^24,25^. Consequently, the Intergovernmental Panel on Climate Change (IPCC) has provided emission factors as a guideline for estimating GHG emissions^26^. The IPCC emission factors were defined for each of the six types of land cover/use categories and nine climate zones, which were divided according to mean annual temperature and potential evapotranspiration. The emission factors have previously been used to estimate GHG emissions through land use changes or forest and peat fires^24,27^. However, some emission factors do not reflect the actual field conditions^25,28–30^. The integrated estimation of the carbon balance between CO_2_ emissions from land use change, fires, and oxidative peat decomposition and CO_2_ uptake by biomass growth is important for understanding carbon cycles in vulnerable tropical ecosystems. However, few studies have simultaneously evaluated both the sources and sinks for CO_2_ in Borneo^31,32^. This study provides a new method for estimating net CO_2_ emissions from AFOLU sectors for the whole of Borneo based on the IPCC guideline with scaling factors found in recent studies (e.g., Borchard et al.^33^ and Basuki et al.^34^ for biomass, Wooster et al.^35^ for fire emissions, and Krisnawati et al.^30^ for peat fire and decomposition emissions). We aimed to quantify all the sources of CO_2_ emissions associated with land use changes, forest and peat fires, and oxidative peat decomposition using satellite data. Accordingly, we (1) mapped annual land use and detected land use changes; (2) estimated CO_2_ emissions through land use changes; (3) estimated CO_2_ emissions from forest and peat fires; (4) estimated CO_2_ emissions from oxidative peat decomposition; and (5) estimated CO_2_ uptake by biomass growth. We created annual land use maps by composite processing to supplement areas of cloud cover, generated features for machine learning to fully utilize multi-band optical data, and processed the data to distinguish mature plantations from forests. We modified the method of CO_2_ emission estimation from biomass burning from Shiraishi et al.^36^ by incorporating belowground biomass (BGB), woody debris, and leaf litter for combustion sources, and using various emission factors for Borneo. CO_2_ emissions from peat fires, oxidative peat decomposition, and CO_2_ uptake from biomass growth were calculated based on the IPCC guidelines with the latest field data from the literature. The results were compared to those of related studies to confirm the validity of our estimates.

## Results

### Land use classification

Annual land use maps from 2000 to 2016 were created using the random forest (RF) classifier. The classification accuracy by RF is shown in Fig. S1. The average accuracy of the land use classification was 85.2 ± 1.5% (± 1 standard deviation), and that for the forest/non-forest classification was 91.4 ± 1.1% (Fig. S1a). The area of no data caused by cloud cover or haze from 2000 to 2016 was 0.44 ± 0.37 Mha year^–1^ (mean ± 1 standard deviation) equivalent to 0.6% of the whole of Borneo. The accuracy of the land use classification in 2016 was 82.0%, which is 2.6–7.0% lower than that in other years outside of the 1 standard deviation. However, the accuracy of the forest/non-forest classification was 90.5% in 2016, which is within 1 standard deviation. These results suggest that, although forest areas could be classified with more than 90% accuracy from 2000 to 2016, misclassifications occurred in the non-forest categories. Regarding producer’s accuracy (Fig. S1b), which indicates the extent of correctly classified supervised data, the classification accuracy was 93.4 ± 0.7% for forest, 87.5 ± 0.8% for shrub/grassland, 80.2 ± 2.4% for urban, and 88.2 ± 1.6% for water. Meanwhile, the accuracy for plantation was relatively low at 21.1 ± 15.2%. However, the user’s accuracy, which indicates the extent of correctly classified data, for plantation was high at 95.7 ± 3.9% (Fig. S1c). These results indicate that areas estimated to be plantations were classified with high accuracy. However, many areas that supervised data showed as plantations were classified into different land use categories. User’s accuracy for shrub/grass was the lowest at 78.0 ± 2.1% within the five categories. In 2000 − 2015, producer’s accuracy for plantations sharply decreased from 58.4% to 6.5%. However, user’s accuracy for shrub/grass decreased from 81.6% to 75.7%, suggesting that many plantation areas were misclassified as shrub/grass. Large-scale deforestation occurred in Borneo between 2000 and 2002^6^. Our results suggested that plantation areas were relatively easily detected immediately after logging. However, vegetation growth makes it difficult to separate them from shrub/grassland or forest.

**Figure 1:**
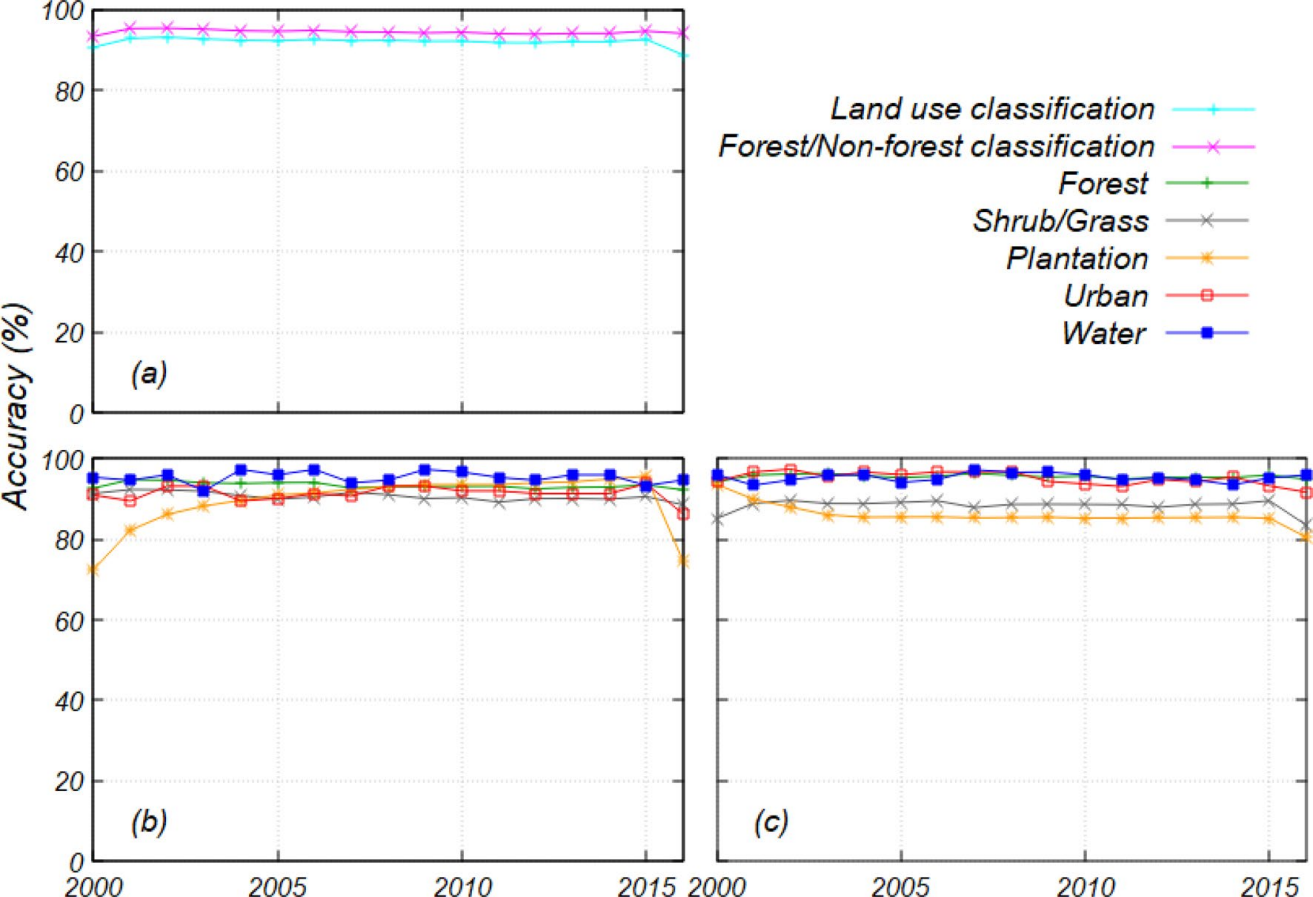
Accuracy of land use maps from 2000 to 2016: (**a**) Overall accuracies for land use and forest/non-forest classification, (**b**) producer’s accuracy for land use classification, and (**c**) user’s accuracy for land use classification.

The accuracies of the land use maps, which were obtained by post-processing after classification (Fig. S2), are shown in Fig. 1. The averages of the overall accuracy improved to 92.0 ± 1.0% for the land use maps and 94.5 ± 0.5% for the forest/non-forest maps (Fig. 1a). The averages for producer’s and user’s accuracy were 93.4 ± 0.7% and 95.6 ± 0.5% for forests, 90.6 ± 1.0% and 88.3 ± 1.6% for shrub/grass, 89.5 ± 6.7% and 86.1 ± 2.6% for plantation, 91.4 ± 1.8 and 95.2 ± 1.6% for urban, and 95.4 ± 1.4% and 95.4 ± 1.1% for water, respectively. The classification accuracy improved in all categories, especially for plantations.

### Land use change

**Figure 2:**
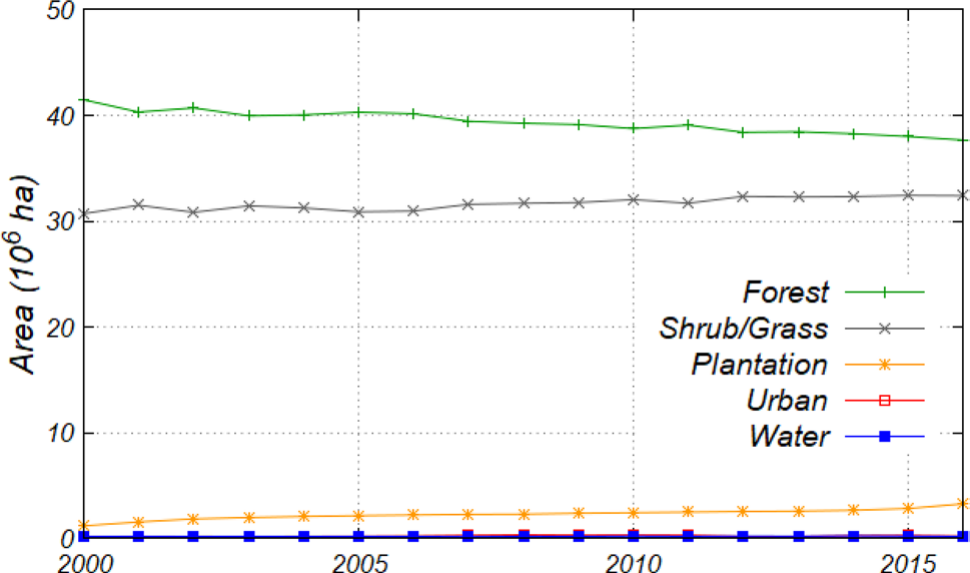
Interannual variation in the area of each land use category from 2000 to 2016.

The annual changes in the area of each land use category are shown in Fig. 2. The forest area in 2000 was 41.5 Mha, covering approximately 56% of Borneo. However, the area decreased to 37.7 Mha, equivalent to 51% of Borneo, in 2016 with a deforestation rate of 0.2 Mha year^−1^ (0.6% year^−1^). The deforestation area from 2000 to 2001 was 1.2 Mha, which was the largest reduction accounting for 30% of the observed deforestation area (3.8 Mha) over the assessment period. Accordingly, the area of the other four land use categories expanded by factors of 1.05 for shrub/grass, 2.74 for plantation, 1.15 for urban, and 1.14 for water from 2000 and 2016. The expansion of water areas was largely derived from the construction of the Bakun and Murum dams in the south-eastern part of Sarawak from 2011 to 2014^10^.

### CO_2_ emissions

**Figure 3:**
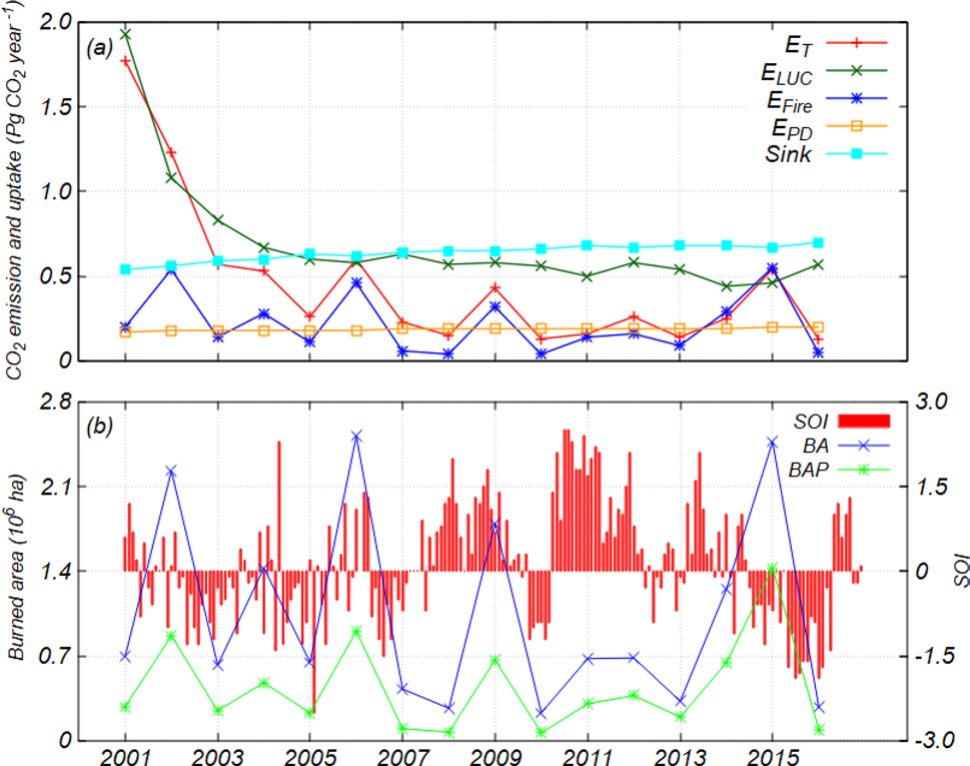
Interannual variation in (**a**) CO_2_ emission and uptake (Pg CO_2_ year^−1^) and (**b**) burned area (10^6^ ha) in Borneo from 2001 to 2016. In (**a**), total net CO_2_ emissions (*E*_T_) are in red, CO_2_ emissions from land use change (*E*_LUC_) are in green, CO_2_ emissions from forest and peat fires (*E*_Fire_) are in blue, CO_2_ emissions from oxidative peat decomposition (*E*_PD_) are in orange, and CO_2_ uptake by biomass growth (*Sink*) is in light blue. *E*_T_ was calculated by subtracting *Sink* from the sum of *E*_LUC_, *E*_Fire_, and *E*_PD_. In (**b**), the Southern Oscillation Index (SOI)^38^ is shown in red bars along with burned areas. The total burned area (BA: blue) and burned peatland area (BAP: green) were detected from the NCM fire map.

The CO_2_ emissions throughout Borneo from 2001 to 2016 are shown in Fig. 3a. CO_2_ emissions were highest in 2001 at 1.77 Pg CO_2_ year^−1^, decreased continuously to 0.26 Pg CO_2_ year^−1^ in 2005, increased in 2006, 2009, 2014, and 2015, and was estimated at 0.13 Pg CO_2_ year^−1^ in 2016, which was the lowest emission rate during the assessment period. The emissions were strongly affected by land use change in 2001 (1.93 Pg CO_2_ year^−1^), 2002 (1.08 Pg CO_2_ year^−1^), 2003 (0.83 Pg CO_2_ year^−1^), and 2004 (0.67 Pg CO_2_ year^−1^), and the emissions were 0.54, 0.46, 0.32, 0.29, and 0.55 Pg year^−1^ from forest and peat fires in 2002, 2006, 2009, 2014 and 2015, respectively. The average CO_2_ emissions from oxidative peat decomposition from 2001 to 2016 were 0.19 ± 0.01 Pg CO_2_ year^−1^ with little interannual variation. The average CO_2_ uptake was 0.64 ± 0.04 Pg CO_2_ year^−1^. These results indicate that high CO_2_ emissions were related to land use change in 2001 and 2002, and fires in 2002, 2006, 2009, 2014, and 2015. WWF^6^ reported a forest loss of 1.3 $\times$ 10^6^ ha year^−1^ in Borneo from 2000 to 2002. We found that the forest area decreased by 1.15 $\times$ 10^6^ ha in 2001 and increased by 0.38 $\times$ 10^6^ ha in 2002. The years of high CO_2_ emissions from fires (i.e. 2002, 2006, 2014, and 2015) corresponded with negative Southern Oscillation Index (SOI) values, and were thus affected by ENSO. Similar to the results of previous studies on large fire events and ENSO in Borneo (e.g., Wooster et al.^21^, Fanin and van der Werf^37^), we observed a significant negative correlation (*R* =  − 0.69, *p* < 0.05) between fire emissions and mean monthly SOI (Fig. 3b). In contrast, CO_2_ uptake through biomass growth across Borneo has shown a significant increasing trend at an annual rate of 1.69% year^−1^.

The average and 1 standard deviation of total net CO_2_ emissions in Borneo from 2001 to 2016 were 461.10 ± 436.51 Tg CO_2_ year^−1^ (Table 1). The interannual variations in CO_2_ emissions and uptake in the eight regions are shown in Figure S3. Regarding the total emissions from 2001 to 2016, the CO_2_ emissions exceeded its uptake in six regions excluding Brunei and North Kalimantan. More than half of the CO_2_ emissions throughout Borneo were emitted from Sarawak (135.02 ± 99.01 Tg CO_2_ year^−1^) and Central Kalimantan (132.50 ± 143.43 Tg CO_2_ year^−1^), accounting for 29.3% and 28.7% of the total emissions for Borneo, respectively. CO_2_ emissions from fires in Sarawak were relatively small and stable compared with those in other regions, whereas those from land use change were the highest at 211.83 ± 87.70 Tg CO_2_ year^−1^. In Central Kalimantan, the CO_2_ emissions from land use change were 119.48 ± 83.92 Tg CO_2_ year^−1^, whereas those from forest and peat fires were the highest at 104.68 ± 105.62 Tg CO_2_ year^−1^, accounting for 48% of the total fire-related emissions in Borneo. Emissions from oxidative peat decomposition were the highest at 55.51 ± 1.76 Tg CO_2_ year^−1^ in Central Kalimantan, accounting for 30% of the total Bornean emissions. This is because the area of peatland is the largest in this region (3.25 Mha) and peat swamp forests have been rapidly degraded^13^. The average emissions from the two Malaysian provinces and the five Indonesian provinces in Borneo were 4.1% and 34.8% smaller, respectively, than the average CO_2_ emissions from fossil fuel, excluding land use change, for each country from 2001 to 2016 based on the Global Carbon Budget 2022^3^. The net CO_2_ emissions per area in peatlands were approximately 3 − 15 times higher than those of mineral soils in all regions (Table 1). These results indicate that forest (aboveground, belowground, debris, and litter) and peat fires in addition to oxidative peat decomposition played major roles in CO_2_ emissions.

**Table 1:** Average and 1 standard deviation of annual CO_2_ emission and uptake with the maximum (Max) and minimum (Min) amounts (Tg CO_2_ year^−1^) for each region from 2001 to 2016.

Region [Peat area/Total area (Mha)]	Total			LUC		Fire		OPD		Sink	
	CO_2_ emissions	Max [Year]	Mineral soil	CO_2_ emissions	Max [Year]	CO_2_ emissions	Max [Year]	CO_2_ emissions	Max [Year]	CO_2_ sink	Max [Year]
		Min [Year]	Peatland		Min [Year]		Min [Year]		Min [Year]		Min [Year]
Borneo	461.10 ± 436.51	1772.30 [2001]	1.46 ± 2.96	695.10 ± 352.25	1932.74 [2001]	216.88 ± 168.14	553.23 [2015]	186.97 ± 7.94	202.35 [2016]	(638.24 ± 44.51)	(696.31) [2016]
[9.78/73.71]		125.71 [2010]	12.93 ± 5.69		444.46 [2014]		39.35 [2010]		174.56 [2001]		(537.97) [2001]
Sabah	54.60 ± 72.19	284.99 [2001]	2.98 ± 5.21	102.59 ± 63.57	313.92 [2001]	6.08 ± 3.88	12.75 [2002]	15.14 ± 0.37	15.69 [2010]	(69.22 ± 9.37)	(80.20) [2014]
[0.68/7.40]		(6.66) [2014]	10.27 ± 2.57		49.96 [2010]		0.99 [2011]		14.3 [2002]		(49.07) [2001]
Sarawak	135.02 ± 99.01	433.08 [2001]	4.29 ± 4.28	211.83 ± 87.70	481.86 [2001]	18.69 ± 7.87	39.22 [2009]	24.66 ± 2.87	27.89 [2016]	(120.16 ± 14.46)	(142.56) [2015]
[1.23/12.38]		(1.79) [2015]	15.42 ± 3.62		100.02 [2015]		6.71 [2003]		19.17 [2001]		(92.97) [2001]
Brunei	(1.25 ± 2.29)	5.93 [2001]	(1.26 ± 1.89)	2.87 ± 1.98	9.31 [2001]	0.22 ± 0.14	0.51 [2003]	0.89 ± 0.03	0.92 [2011]	(5.24 ± 0.38)	(5.83) [2015]
[0.08/0.58]		(3.45) [2016]	(0.14 ± 2.69)		1.19 [2016]		0.01 [2006]		0.83 [2001]		(4.62) [2001]
North Kalimantan	(13.20 ± 20.9)	61.26 [2001]	(1.51 ± 1.45)	33.80 ± 20.37	106.89 [2001]	2.42 ± 1.69	5.58 [2004]	10.34 ± 0.45	11.16 [2016]	(59.76 ± 0.86)	(60.98) [2015]
[0.62/7.01]		(30.54) [2006]	4.70 ± 3.04		18.73 [2006]		0.47 [2011]		9.72 [2002]		(57.25) [2001]
East Kalimantan	43.85 ± 50.68	197.92 [2001]	0.81 ± 1.98	99.54 ± 38.98	236.85 [2001]	20.36 ± 18.42	75.83 [2015]	24.14 ± 0.26	24.72 [2015]	(100.18 ± 8.49)	(111.63) [2016]
[1.08/12.57]		(1.48) [2005]	10.89 ± 4.34		63.51 [2005]		2.98 [2008]		23.79 [2005]		(81.89) [2001]
West Kalimantan	67.90 ± 68.94	271.26 [2001]	0.72 ± 2.38	99.79 ± 58.54	301.95 [2001]	40.68 ± 29.48	91.79 [2006]	36.71 ± 2.87	41.87 [2016]	(109.28 ± 5.29)	(115.94) [2015]
[2.12/14.67]		1.60 [2007]	10.92 ± 4.86		64.05 [2006]		4.47 [2010]		33.31 [2001]		(97.65) [2001]
Central Kalimantan	132.50 ± 143.43	432.39 [2001]	0.99 ± 3.58	119.48 ± 83.92	417.53 [2001]	104.68 ± 105.62	330.43 [2002]	55.51 ± 1.76	60.69 [2016]	(147.17 ± 8.19)	(157.38) [2013]
[3.25/15.36]		(14.93) [2008]	14.96 ± 11.55		63.26 [2014]		0.79 [2010]		53.33 [2002]		(130.29) [2001]
South Kalimantan	41.69 ± 32.81	113.49 [2002]	1.89 ± 3.66	25.21 ± 18.99	70.57 [2002]	24.13 ± 18.6	66.74 [2015]	19.58 ± 0.15	19.78 [2015]	(27.24 ± 2.36)	(30.89) [2016]
[0.72/3.75]		0.52 [2016]	19.65 ± 9.26		9.59 [2008]		0.47 [2010]		19.12 [2001]		(23.26) [2002]

The total net CO_2_ emissions (Total) were calculated by subtracting the CO_2_ uptake from biomass growth (Sink) from the sum of the emissions through land use change (LUC), forest and peat fires (Fire), and oxidative peat decomposition (OPD). The total net CO_2_ emissions per mineral soil and peatland area (Mg CO_2_ year^−1^ ha^−1^) are shown for the total CO_2_ emission/uptake.

The values in parentheses represent net CO_2_ uptake.

## Discussion

### Land use change

The average accuracies from 2000 to 2016 were 92.0 ± 1.0% for land use maps and 94.5 ± 0.5% for forest/non-forest maps. The deforestation rate of 0.6% year^−1^ from 2000 to 2016 was smaller than that in previous studies (Table S1) but comparable to that reported by Gaveau et al.^10^. The estimated forest areas were 1.0% smaller in 2000 and 1.7% larger in 2015 compared to those reported by Gaveau et al.^10^, who used manually created maps after automatic land use classification. According to the country reports from 2020 of the Global Forest Resources Assessments, domestic deforestation rates for Indonesia and Malaysia from 2000 to 2016 were 0.31% year^−1^ (FAO^39^) and 0.01% year^−1^ (FAO^40^), respectively. Hansen et al.^41^ reported a deforestation rate of 0.60% throughout Indonesia from 2000 to 2005. Therefore, our estimated deforestation rates for Borneo correspond closely to previously reported results. However, the plantation areas in this study were 67.0% and 69.5% smaller than those reported by Gaveau et al.^10^ in 2000 and 2015, respectively. The land use maps of Gaveau et al.^10^ included oil palm plantation areas that could not be identified as plantations by visual interpretation using Google Earth™. Gaveau et al.^10^ potentially considered forest areas before development and partially logged areas, such as grassland and bare land, as plantation areas, or we were unable to identify plantation areas immediately after planting due to the resolution of Google Earth™ data. The supervised data for forest and shrub/grassland in this study were frequently scattered in plantation areas in Gaveau et al.^10^. The accuracy of the forest/non-forest maps of each study in Table S1 was over 90%. However, the deforestation rates were different. This may have been predominantly influenced by the training data for land use mapping and the validation data for evaluation. Further collection of land cover/use information for supervised data is needed to improve the quality of the maps.

### CO_2_ emissions from land use change

The CO_2_ emissions from land use changes in forest areas were compared with data from the Indonesian National Carbon Accounting System (INCAS^24,32^) (Table S2). The average annual CO_2_ emissions (Eavg) were greater than those in the INCAS in every region. The CO_2_ emissions (Emin) in each region excluding North Kalimantan were greater than those in the INCAS. This finding suggests that the INCAS estimates were smaller than the minimum CO_2_ emissions estimated in all candidate coefficients. Furthermore, the areas converted from forest to non-forest in this study were larger in all provinces than those in the INCAS (Fig. S4). The area of land use change was larger in this study than in the INCAS by factors of 1.2 in North Kalimantan to 4.1 in South Kalimantan. However, the deforestation rate in this study was the lowest among the previous studies (Table S1). The deforestation area from 2000 to 2015 reported by Gaveau et al.^10^, which was the second lowest deforestation rate among the compared studies, was estimated at 4.7 $\times$ 10^6^ ha, which was 26.4% larger than that of this study. The INCAS reported that the total forest area increased by 9.7% from 2001 to 2012 in five provinces of Indonesian Borneo^32^. One explanation for the difference in CO_2_ emissions from land use change between this study and the INCAS could be the difference in the estimation of the forest area.

### CO_2_ emissions from forest and peat fires

The CO_2_ emissions from fires in this study were compared with the Global Fire Emissions Database Version 4.1 with small fires (GFED4.1s^42^) (Table S3). The average annual CO_2_ emissions (Eavg) in Borneo from 2001 to 2016 were 217.27 ± 166.85 Tg CO_2_ year^−1^ in this study, which was 18.4% larger than those in the GFED4.1 s. The CO_2_ emissions (Eavg) in each region excluding South Kalimantan were larger than those in the GFED4.1 s. Although the CO_2_ emissions (Emin) in three regions (Sabah, Sarawak, and North Kalimantan), were relatively low compared with those in the other regions, they were larger than those in the GFED4.1 s. GFED4.1 s emissions in the other six regions including the whole of Borneo were within the Emax and Emin range. Fire-related CO_2_ emissions in Borneo in our previous study^36^, which used similar methods to this study with only aboveground biomass (AGB) for burned biomass, were 182.16 ± 140.69 Tg CO_2_ year^−1^. CO_2_ emissions estimated by adding BGB, WDL, and peat fires were 16.2% greater than those reported by Shiraishi et al.^36^, thus differing greatly from the emission data in the GFED4.1 s. Carbon monoxide (CO) is often used to assess fire emissions because its lifespan in the atmosphere is weeks to months^43,44^. Saito et al.^44^ evaluated the atmospheric CO concentrations obtained by simulating atmospheric CO variability using an atmospheric tracer transport model and fire CO emissions from GFED4.1 s in comparison with satellite observation data from the Measurements of Pollution in the Troposphere (MOPITT). Consequently, the GFED4.1 s emissions were 17 − 31% lower than those of the satellite data. Notably, CO_2_ emissions, which were estimated using the same method, could also be approximately 30% lower. Wooster et al.^35^ reported CO_2_ emissions of 692 ± 213 Tg released from large fires in Indonesian Borneo during September to October 2015, predominantly due to drying caused by an El Niño event. These emissions were higher than the estimated 530.73 Tg CO_2_ year^−1^ for 2015 in this study. Wooster et al.^35^ applied the scaling parameters obtained based on the 2015 El Niño event, which may have contributed to the difference in our estimates. Although MOD14A1 for fire distribution and frequency provides daily fire areas from 2001, the fire detection rate was 82%^45^. Smaller areas of fire can be overlooked, especially in areas of frequent cloud cover, such as Southeast Asia.

CO_2_ emissions from peat fire were compared with data from the INCAS (Table S4). The total annual CO_2_ emissions (Eavg) in this study for Indonesian Borneo from 2001 to 2012 were 124.37 ± 104.73 Tg CO_2_ year^−1^, approximately three times larger than those in the INCAS, which was 33.67 ± 31.07 Tg CO_2_ year^−1^. The CO_2_ emissions (Eavg) were greater than those in the INCAS in all regions (by factors of 3.1 for Central Kalimantan to 40.5 for North Kalimantan). The CO_2_ emissions (Emin) in every region were greater than those in the INCAS. The peat area, which is the basis for evaluation, differed between this study and the INCAS (Table S5). The total peat area in this study^46^ was approximately 11 times larger than that for the INCAS. One reason for the difference in fire emissions could be related to differences in fire frequency and input parameters. Peat fire emissions are generally higher for the first fire than the second fire in the same area because of a decrease in the burn depth^25^. Although we calculated the number of fires since 2001, the INCAS considers fires that occurred prior to 2001. In the INCAS estimates, the peat area for the first fire was 12 ha, while the peat area for the second fire was 45 301 ha in Central Kalimantan. Therefore, the emissions in this study were calculated from a greater burned depth than those by the INCAS. Differences in peat area have also contributed to differences in emission estimates. The peat distribution map of Gumbricht et al.^46^ potentially included rice paddies and other fields in the peat areas, which were estimated to be approximately three times larger than those of Page et al.^12^ and 41.6% larger than that estimated by Anda et al.^47^. Therefore, compared to the INCAS, more fires may have been assessed as peat fires in estimating emissions from peat fires. The peat area also had a significant impact on the estimation of CO_2_ emissions from oxidative peat decomposition. Using accurate peat distribution maps and determining input parameters that account for fire intensity is important to improve the accuracy of CO_2_ emission estimation.

### CO_2_ emissions from oxidative peat decomposition

The CO_2_ emissions from oxidative peat decomposition were compared with those in the INCAS (Table S6). The total annual CO_2_ emissions (Eavg) in Indonesian Borneo from 2001 to 2012 were 143.87 ± 2.82 Tg CO_2_ year^−1^, 31.4% larger than those in the INCAS. Eavgs were 0.4% larger for Central Kalimantan and 4.4% smaller for West Kalimantan than those in the INCAS. Eavgs in North Kalimantan, East Kalimantan, and South Kalimantan were 2.7 to 19.3 times higher than those in the INCAS, and each value in the INCAS was lower than the Emins in the three regions.

The emission factors also affect the difference in estimated CO_2_ emissions between this study and the INCAS. We used emission factors based on the IPCC guidelines, whereas the INCAS uses different emission factors depending on whether drainage occurred within the last five years or more^48^. Based on this limitation, future studies could benefit from determining the drainage area distribution and timing of drainage, and evaluating temporal changes in emission factors.

### CO_2_ sink

The CO_2_ sink for forest areas was compared with data from the INCAS (Table S7). The total annual CO_2_ sink (Eavg) in Indonesian Borneo from 2001 to 2012 was 283.13 ± 18.42 Tg CO_2_ year^−1^, over 100 times larger than that in the INCAS, which was 2.68 ± 1.18 Tg CO_2_ year^−1^. The CO_2_ sinks in the forest, shrub/grass, and plantation land use categories were 419.06 ± 35.10, 202.73 ± 9.13, and 17.61 ± 3.42 Tg CO_2_ year^−1^, respectively, suggesting that biomass growth in the vast forests of Borneo contributed to CO_2_ uptake. The CO_2_ sinks (Emin) in every region were greater than those in the INCAS. Furthermore, the CO_2_ sink throughout Indonesian Borneo was 438.60 ± 23.04 Tg CO_2_ year^−1^, which is 1.5 times larger than the uptake from forest areas. The main reason for the difference in estimates is biomass growth. Although we determined biomass growth for each land use category based on the latest studies, the INCAS assumes no net growth in primary forests, that is, growth is equivalent to turnover and decomposition^24^, which may underestimate CO_2_ sink effects. The vast area of intact forest in Borneo is considered to be an important carbon sink. For example, Pan et al.^49^ estimated an average carbon storage rate of 0.90 Mg C ha^−1^ year^−1^ in tropical intact forests in Asia, and the IPCC^26^ estimated an average carbon storage rate of 3.4 t dry matter ha^−1^ year^−1^ in tropical rainforests over 20 years in Asia. The CO_2_ sink throughout Malaysian Borneo in 2014 was estimated at 213.25 Tg CO_2_ year^−1^, which is equivalent to 80% of the estimate for the whole of Malaysia (267.17 Tg CO_2_ year^−1^)^50^. The forest areas of Malaysian Borneo were 1.5 times larger than those of Peninsular Malaysia^50^, and the CO_2_ sink from non-forest areas (shrub/grass and plantation) across Malaysian Borneo accounted for 34% of the CO_2_ sink from forests in the region. Along with vast forest areas, regrowth from deforested and disturbed areas and growth of young plants through plantation management are considered to have contributed to the expanding CO_2_ sink.

### Directions for further research

The supervised data for land use classification and scaling factors for emission estimation considerably affected the accuracy of our results. The forest area in this study effectively captured the characteristics of the supervised data, with a classification accuracy of over 94%, consistent with the results of Gaveau et al.^10^. However, the plantation area needs further evaluation. Future studies should further evaluate features that contribute to the classification accuracy of plantations, such as oil palm and pulpwood, and collect information on plantation areas that are difficult to evaluate visual interpretation of Google Earth™ maps alone. Although the method for estimating CO_2_ emissions in this study made extensive use of scaling factors based on values from the literature and IPCC guidelines, further investigations are needed. BGB, woody debris, and leaf litter were calculated using conversion factors from AGB. However, if AGB decreases due to deforestation, woody debris and leaf litter would increase^51^. Biomass growth was determined in each land use category, but is expected to vary by site, tree species, and fire prevalence. Although we determined biomass growth from values in the literature for several sites of Borneo, the values do not cover all of these conditions. More accurate and frequent biomass estimation is needed (e.g., Hayashi et al.^52^). Although scaling factors of BE and EF were commonly used for AGB, BGB, and WDL to estimate the biomass burning emissions, the values may differ in the burned part. For example, the BE of AGB and WDL is expected to be greater than that of BGB. Accordingly, it could be useful to develop a fire detection algorithm incorporating MODIS, which comprises long-term daily observations, and newer satellite data (e.g. Roy et al.^53^) to improve the fire detection rate. Furthermore, fire emissions vary with fire intensity. Determining the scaling factors for these environments and conditions and incorporating them into the model are important considerations for future work. The integrated use of various types of datasets, such as Global Ecosystem Dynamics Investigation (GEDI) data, which provides the canopy height and vertical structure of forests, P-band SAR data, which is planned to be launched in 2024 by the European Space Agency, quality improvement of supervised data and field observation data, and continuous development of estimation models are essential for improving estimation accuracy.

## Conclusions

In Southeast Asia including Borneo, land use changes, particularly the conversion of peat swamp forests to plantations, may have an important impact on climate change. This study presents a method for estimating CO_2_ emissions from land use changes, fires, and oxidative peat decomposition in Borneo from 2001 to 2016 and compared and evaluated the estimated results with those in previous studies. The model was developed with CO_2_ uptake from biomass growth and CO_2_ emission estimation in non-forested areas, which had not been evaluated in previous studies. The CO_2_ sink from biomass growth increased significantly in all regions including the whole of Borneo. However, the net CO_2_ balance showed that emissions exceeded uptake. Furthermore, the estimated CO_2_ emissions from land use change, fires, and oxidative peat decomposition tended to be larger than those in previous studies. These results suggest that higher levels of CO_2_ have been emitted than previously estimated. Our method for estimating CO_2_ emissions can contribute to the establishment and improvement of forest management systems for countries aiming to expand their forest areas and enhance forest management.

Satellite remote sensing is an important tool for estimating GHG budgets in a rapidly fluctuating environment of tropical rainforests, where regular field measurements are difficult. However, there is room for research to improve estimation accuracy. Flux data obtained from field surveys in a variety of environments, supervised data for land use classification in which discrepancies were with previous studies, and improvements in peat distribution maps and burned area detection over time are essential for progress in this field. Furthermore, the application of new satellite observation datasets and existing resources to generate biomass maps as a basis for CO_2_ uptake and emission is an important future task.

## Materials and methods

### Study area

**Figure 4:**
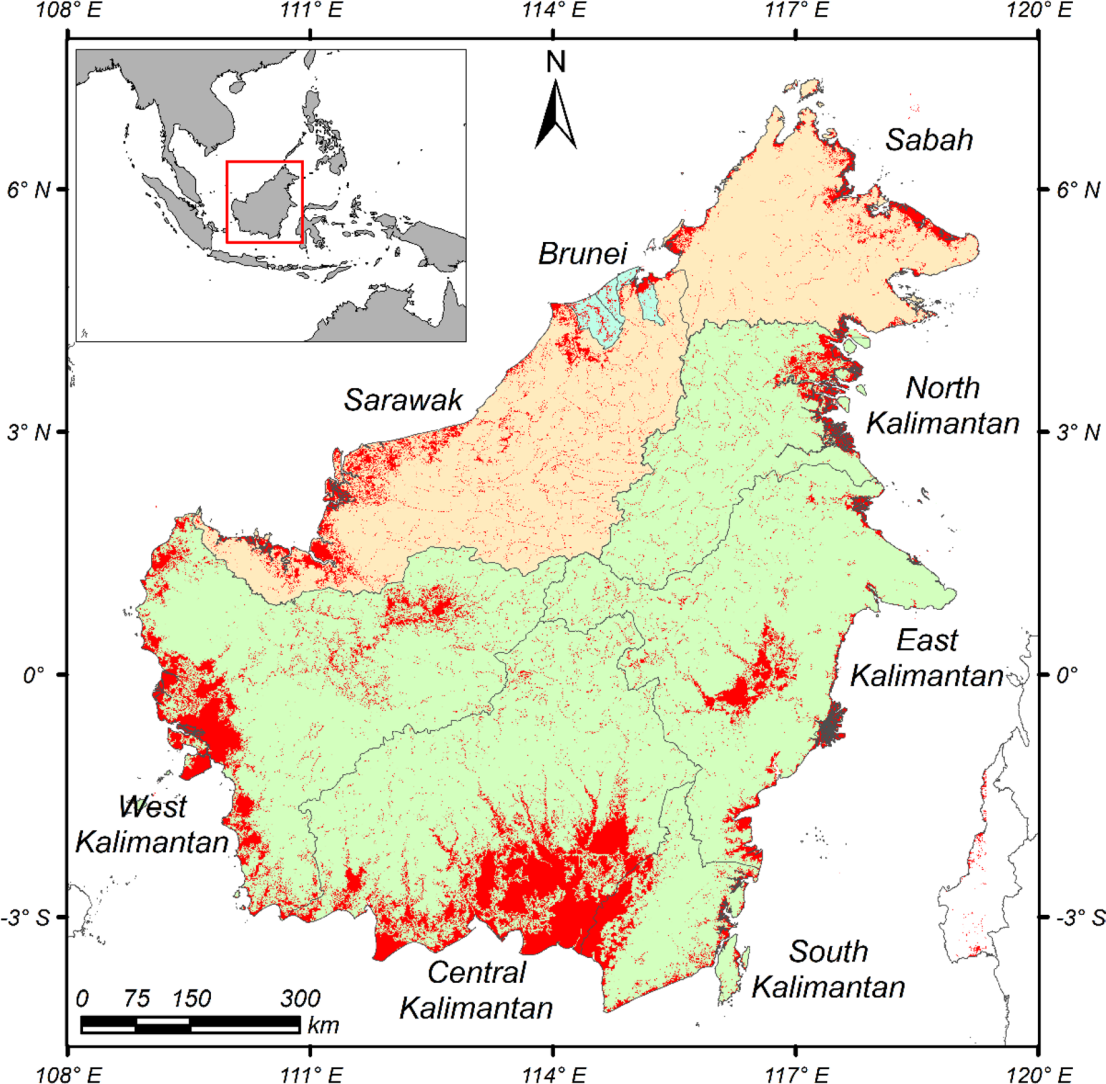
Map of Borneo. The eight regions assessed were Sabah and Sarawak in Malaysia; Brunei Darussalam; and Northern Kalimantan, East Kalimantan, West Kalimantan, Central Kalimantan, and South Kalimantan in Indonesia. The area of peat is shown in red (Gumbricht et al.^46^). Maps were created with ArcMap version 10.5 (https://www.arcgis.com/).

Borneo is the third largest island (74.3 Mha) worldwide and is shared by Indonesia, Malaysia, and Brunei Darussalam (Fig. 4). The island is located between 8°N and 5°S across the equator and between 108°E and 120°E in a tropical rainforest climate (according to the Köppen climate classification). In this region, the wet northeast monsoon prevails from November to March or April, while the prevailing wind changes to the dry southwest monsoon in May–June and September–October^9^. The ENSO causes delayed onset of the rainy season, reduced precipitation, and prolonged dryness in the region^19–21^. Although Borneo has the largest forest area in Southeast Asia and is rich in biodiversity, it is also a major hotspot for deforestation, which has predominantly been driven by fires and land conversion into plantations^54^. There are 9.8 Mha of peatlands, accounting for 33% of those in Southeast Asia^46^. By 2010, 13% of peatlands had already been converted into industrial plantations, such as for oil palm and pulpwood^55^. Inland forests with abundant biomass and peat swamp forests with high levels of peat carbon have become the main sources of CO_2_ emissions from land use change through agricultural development^10^, burning of wood materials^21^, and drying peat from drainage^22,23^.

### Data

#### Remote sensing data

The National Aeronautics and Space Administration (NASA) Moderate Resolution Imaging Spectroradiometer (MODIS) MCD43A4 Version 6 product was used to create annual land use maps from 2000 to 2016. MCD43A4 has seven-band datasets based on the Nadir Bidirectional Reflectance Distribution Function (BRDF) Adjusted Reflectance (NBAR) with a spatial resolution of 4.18 $\times$ 10^−3^ degrees (approximately 463 m) and daily observation frequency. MCD43A4 also has data quality information for all the grids in each band.

Annual maps of land use, fire, and AGB and a peat distribution map were used to estimate CO_2_ emissions from forest and peat fires for 2001 to 2016. Land use maps were created from MCD43A4. Fire maps were created using the MODIS Thermal Anomalies and Fire (MOD14A1) Version 6 product to estimate the annually burned areas and number of fires. MOD14A1 is a global fire dataset with a 1-km spatial resolution and daily observation frequency^56,57^. Each fire pixel is assigned as having low (0–30%), nominal (30–80%), or high (80–100%) confidence levels^58^. Two types of fire maps were created using nominal confidence maps, that is, NCM with nominal and high confidence levels, 30% to 100% reliability, and low confidence maps (LCM) with low, nominal, and high confidence levels of 0% to 100% reliability. We counted the daily number of fire pixels on the fire maps for each year. An ongoing fire located in the same grid position was considered to be a single fire^36,59^.

For the AGB maps, GEOCARBON and Globbiomass were used. GEOCARBON is a global AGB map with a 1-km spatial resolution for 2010^60^. Globbiomass is also a global AGB map with a 25-m spatial resolution for 2000^61^. The two AGB products were updated annually by calculating the biomass change from land use changes, fires, and biomass growth. The peat distribution map Tropical and Subtropical Wetlands Distribution version 2^46^ was used to estimate the emissions from 2001 to 2016 from peat fires and oxidative peat decomposition.

#### Supervised data for land use classification

Supervised data (training and validation data) from 2000 and 2016 were created by visual interpretation using Google Earth™ for land use classification (Figure S5). The supervised data included point data for the five land use categories, namely forest, shrub/grass, plantation, urban, and water. The supervised datasets included 6668, 6669, and 5839 datapoints in 2000, 2016, and each year from 2001 to 2015, respectively (Table S8). Supervised data were created in the same position (grid) on the map in 2000 and 2016. Assuming that land use did not change if the land use categories at the same position were unchanged in 2000 and 2016, grid data with the same land use categories in the two years were only used for the land use classification from 2001 to 2015. Grid data with different categories between 2000 and 2016 were used to create the land use maps for 2000 and 2016, respectively.

### Land use classification and land use change detection

#### Features for machine learning

The following 228 features were used as input data for machine learning:

1. Seven band reflectance values of MCD43A4, that is, band1 through band7.
2. 13 indices calculated from the seven bands of MCD43A4 (Table S9).
3. Time series data: Time series values obtained from the daily MCD43A4 data over a 1-year period (Table S10). Eight types of time series data were calculated for each of the seven bands.

For each of the 76 features (i.e. 7 bands + 13 indices + 8 time series data × 7 bands), the average and 1 standard deviation in a 9 $\times$ 9 window were calculated to obtain the surrounding information for the target grid.

#### Calculation of land use change areas

Land use change areas for each land use category were calculated from the annual land use maps from 2000 to 2016 created using the RF classifier (Fig. S2). RF is one of the most accurate classifiers for land use classification (e.g., Shiraishi et al.^62^).

Daily MCD43A4 data were composited to create an annual dataset, based on the latest land cover information for each year (December 31 observation). Data for low-quality grids (affected by cloud and haze effects) were replaced with the latest data without quality problems for the year. However, if high-quality data were not available for the year, these grids were excluded from the classification process. After the classification process, the land use category for empty grids was determined based on the surrounding grids.

The land use classification was performed 5 times separately for each grid using the RF approach with supervised data divided into five equal-sized groups. The final category was then determined based on the most frequent category in the five-fold classification. If the frequency was the same, the more frequent land use category in the supervised data was selected. Once a grid had been classified as a plantation, the grid did not change thereafter into any other category. Given that it is almost impossible for urban and water regions to be converted into forest in one year, the classification results were changed to shrub/grass instead of forests. Land use change areas were calculated for each land use category comparing two consecutive land use maps per year.

### Estimation of CO_2_ emissions

The methodology for CO_2_ emission estimation from land use change, forest and peat fires, and oxidative peat decomposition is shown in Fig. S6. The AGB map products GEOCARBON and Globbiomass were created based on observations for 2000 and 2010, respectively. For this study, AGB in 2000 was used to estimate annual CO_2_ emissions from 2001 to 2016. Therefore, the AGB map from 2000 for Globbiomass was created by subtracting the amount of AGB growth from 2000 to 2010 from Globbiomass 2010. The AGB growth for the 10 years was estimated using the annual AGB growth (Table S11) under the assumption that the error of the AGB maps was smaller than the AGB growth over 10 years. The AGB maps were updated annually because of the annual increase in growth and decrease in land use change and fires. We used two fire maps (NCM and LCM) and two AGB maps (GEOCARBON and Globbiomass). Since biomass burning causes a decrease in AGB, fire maps of two types with different burning distributions were generated for the different AGB maps with different distributions. Therefore, four different estimates of CO_2_ emissions were obtained for the two fire and two AGB maps and the final emissions were averaged.

The total net CO_2_ emissions (*E*_T_: g CO_2_ year^−1^) were calculated by subtracting CO_2_ uptake (*Sink*: g CO_2_ year^−1^) by biomass growth from the sum of emissions from land use change (*E*_LUC_: g CO_2_ year^−1^), biomass burning (*E*_BB_: g CO_2_ year^−1^), peat burning (*E*_PB_: g CO_2_ year^−1^), and oxidative peat decomposition (*E*_PD_: g CO_2_ year^−1^), as in Eq. (1).

1 $${E}_{T(p)}= {E}_{LUC(p)}+{E}_{BB(p)}+{{E}_{PB(p)}+E}_{PD(p)}- {Sink}_{\left(p\right)} ,$$

where *p* is the grid position for the calculation target.

2 $${E}_{LUC\left(p\right)}= {CA}_{\left(p\right)}\left({D}_{AGB\left(p\right)}\cdot {CC}_{L}+{D}_{AGB\left(p\right)}\cdot {R}_{BGB}\cdot {CC}_{L}+{D}_{AGB\left(p\right)}\cdot {R}_{WDL}\cdot {CC}_{D}\right){C}_{CtoCO2},$$

3 $${D}_{AGB(p)}= {AGB}_{pre\left(p\right)}-{AGB}_{cur\left(p\right)}.$$

Annual AGB growth (t dry matter ha^−1^ year^−1^) and AGB change associated with land use change are defined in Table S12. BGB (t dry matter ha^−1^) and the sum of woody debris and leaf litter (*WDL*: t dry matter ha^−1^) were calculated from AGB using conversion factors (%) (Table S13). The first term of the right side in Eq. (2) refers to the carbon loss from AGB by land use change. Emissions from the carbon loss were calculated by multiplying the difference in AGB (*D*_AGB_: t dry matter ha^−1^) before (*AGB*_pre_) and after land use change (*AGB*_cur_), as in Eq. (3), using the biomass carbon content for living trees (*CC*_L_: %). The second term refers to carbon loss from BGB, calculated as the product of *D*_AGB_, conversion factors (*R*_BGB_: %) (Table S13), and *CC*_L_. The third term refers to carbon loss from WDL, calculated as the product of *D*_AGB_, conversion factors (*R*_WDL_: %) (Table S13), and carbon content of dead wood (*CC*_D_: %). *CC*_L_ and *CC*_D_ were set at 45.60%^28^ and 47.16%^29^, respectively. Finally, *E*_LUC_ was calculated by multiplying the land use change area (*CA*: ha) with the sum of the first to third terms and a conversion factor from carbon to CO_2_ (*C*_CtoCO2_: 44/12).

*E*_BB_ was calculated by multiplying the burned area (*BA*: m^2^) with the total biomass density (*BD*: kg m^−2^), burning efficiency (*BE*), and an emission factor (*EF*: g CO_2_ kg^−1^) (Eq. (4)). However, this equation does not consider multiple fires that occurred in one year. Therefore, we evaluated the total AGB density (*BD*_AGB_) considering multiple fires over a year using Eq. (5) to determine the *BD*_AGB_ in Eq. (4)^36^.

4 $${E}_{BB\left(p\right)}= {BA}_{\left(p\right)}\left({BD}_{AGB\left(p\right)}\cdot {BE}_{\left(c\right)}\cdot {EF}_{\left(c\right)}+{BD}_{AGB(p)}\cdot {R}_{BGB}\cdot {BE}_{(c)}\cdot {EF}_{(c)}+{BD}_{AGB\left(p\right)}\cdot {R}_{WDL}\cdot {BE}_{(c)}\cdot {EF}_{(c)}\right),$$

5 $${BD}_{AGB(p)}={\sum }_{i=1}^{I}\left\{{AGB}_{pre(p)}\cdot {\left(1-{BE}_{(c)}\right)}^{i-1}\right\},$$

where *I* is the number of fire occurrences in the target year, and *c* represents the land use category in the fire area. The first term of the right side in Eq. (4) refers to CO_2_ emissions from AGB burning, which was calculated by multiplying *BD*_AGB_ with *BE* and *EF*. *BE* and *EF* were defined for each land use category (Table S14). The second and third terms represent CO_2_ emissions from BGB and WDL burning, respectively. Finally, *E*_BB_ was calculated by multiplying *BA* with the sum of the first to third terms.

*E*_PB_ was calculated by multiplying *BA* with burned peat depth (*BurnD*: m), decreasing rate (*DR*: 0 to 1) of *BurnD*, peat bulk density (*BulkD*: g m^−3^), *BE*, and *EF* (Eq. (6)).

6 $${E}_{PB\left(p\right)}= {BA}_{\left(p\right)}\cdot  BurnD \cdot  DR \cdot {BulkD}_{(c)}\cdot {BE}_{(c)}\cdot {EF}_{(c)}.$$

*BurnD* was determined for the first fire (Table S15). However, *BurnD* decreases with repeated fires^25^. Therefore, *DR* was determined as the ratio of the burned depths^25^ (Table S16). *BulkD* was determined for each land use category (Table S17).

*E*_PD_ was calculated by multiplying the peatland area (*PA*: ha) with *EF* (Table S18) defined for each land use category based on annual land use maps and the peat map (Eq. (7)).

7 $${E}_{PD\left(p\right)}= {PA}_{\left(p\right)}\cdot  {EF}_{(c)}.$$

CO_2_ emissions depend greatly on the drainage of peat. Three non-forest categories (shrub/grass, plantation, and urban) and forests that changed to non-forest at least once on peat areas from 2000 to 2016 were regarded as drained peat areas.

CO_2_ uptake by biomass growth (*Sink*) was calculated by multiplying the biomass growth in each land use category (Table S11) with its area.

### Evaluation

Five-fold cross validation was applied to evaluate the overall, user’s, and producer’s accuracies of the RF-based land use classification, including the five categories forest, shrub/grass, plantation, urban, and water, and forest/non-forest classification, including the four categories shrub/grass, plantation, urban, and water. Plantation areas were carried over and changes in the classified categories from forests to grass/shrub areas were conducted in post-processing after classification (Fig. S2). Therefore, apart from the classification accuracy, the final land use maps were compared to the supervised data to determine their accuracy.

INCAS provides the annual GHG emissions of forest and peat areas in Indonesia from 2001 to 2012^32^. The Full Carbon Accounting Model^63^ has been used to estimate GHG emissions. Although the scaling factors for estimating GHG emissions were based on the IPCC guidelines^26,64,65^, some were collected from various agencies in Indonesia. The forests have been broadly divided into dry forests and wetland forests, which are further divided into primary and secondary forests. Meanwhile, non-forest areas were not separated into agricultural lands or urban areas. Biomass growth was not considered, and all the deforested areas were assumed to be converted into agricultural land. The CO_2_ emissions data from our study were compared with those for INCAS to crosscheck the five provinces in Indonesian Borneo. CO_2_ emissions from forest and peat fires over Borneo were compared to GFED4.1s^42^.

We used various field data from previous studies to determine the scaling factors. To evaluate the uncertainty resulting from input parameters and the difference in estimated CO_2_ emissions between our results and those from previous studies, three types of CO_2_ emissions were calculated, namely (1) CO_2_ emissions (Eavg) estimated using the input parameters explained above, and (2) Emax and (3) Emin estimated using the maximum and minimum values, respectively, of each candidate input parameter.

### Supplementary Information

Supplementary Information.

## Data Availability

The datasets used and analysed in the current study are available from the corresponding author on reasonable request.
