# Supplementary material for: Carbon dioxide emissions through land use change, fire, and oxidative peat decomposition in Borneo
Source: Sci Rep. 2023 Aug 11;13:13067. doi: 10.1038/s41598-023-40333-z (PMC10421864; doi:10.1038/s41598-023-40333-z)
Supplement: Supplementary file 1 — Supplementary Information. [file 41598_2023_40333_MOESM1_ESM.pdf]

Supplementary Materials for

Carbon dioxide emissions through land use change, fire, and peat oxidative decomposition in Borneo

Tomohiro Shiraishi[a,b]*, Ryuichi Hirata[a], Masato Hayashi[c], Takashi Hirano[d]
Correspondence to: shiraishith@nbu.ac.jp

[a]Earth System Division, National Institute for Environmental Studies, Ibaraki 305-8506, Japan
[b]School of Engineering, Nippon Bunri University, Oita 870-0397, Japan
[c]Earth Observation Research Center, Japan Aerospace Exploration Agency, Ibaraki 305-8505, Japan
[d]Research Faculty of Agriculture, Hokkaido University, Hokkaido 060-8589, Japan

This file includes:

Figures: S1 to S6
Tables: S1 to S18
References

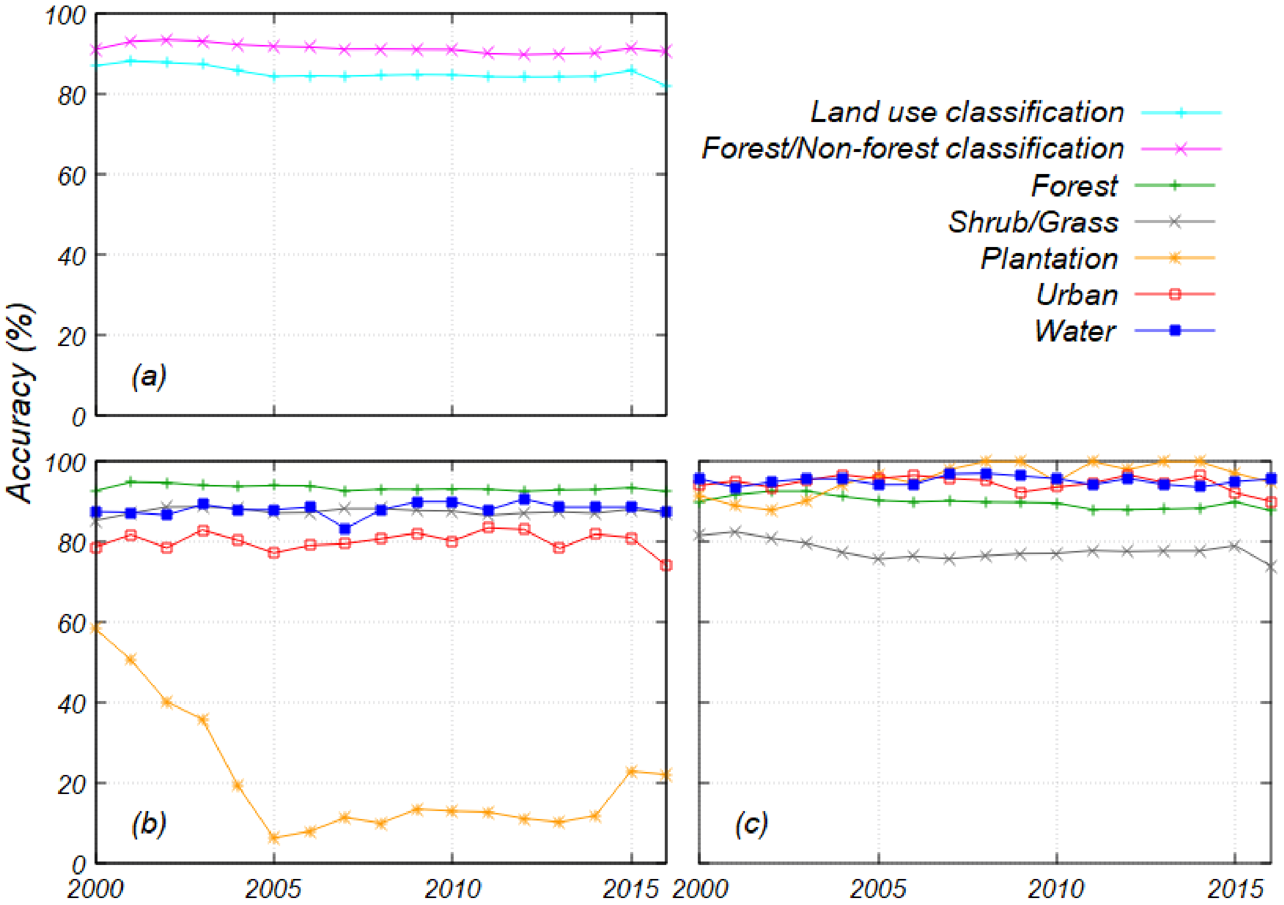

Figure S1. Accuracy of land use and forest/non-forest classifications from 2000 to 2016: (a) overall accuracies for land use and forest/non-forest classification, (b) producer's accuracy for land use classification, and (c) user's accuracy for land use classification.

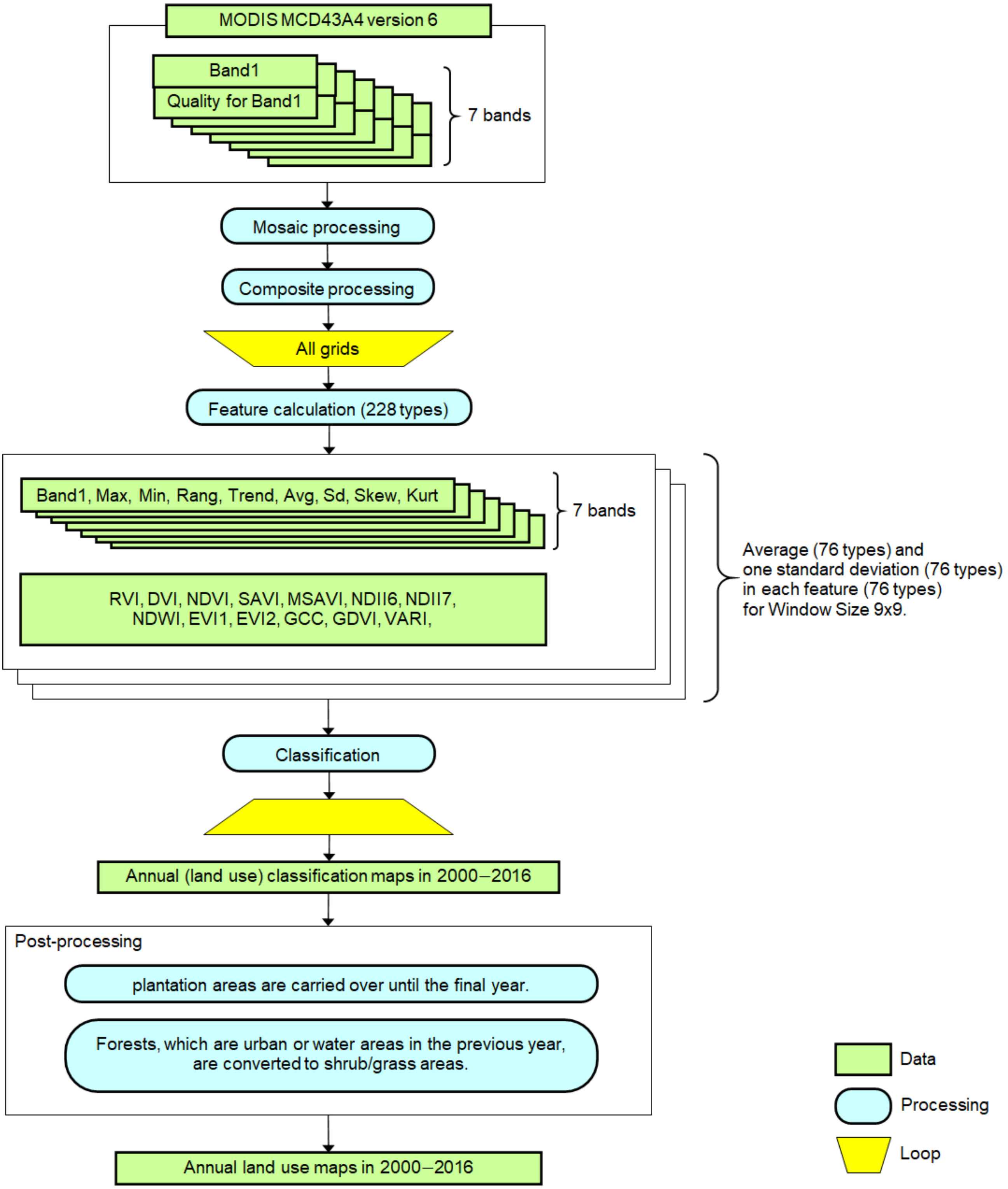

Figure S2. Processing procedure for land use mapping.

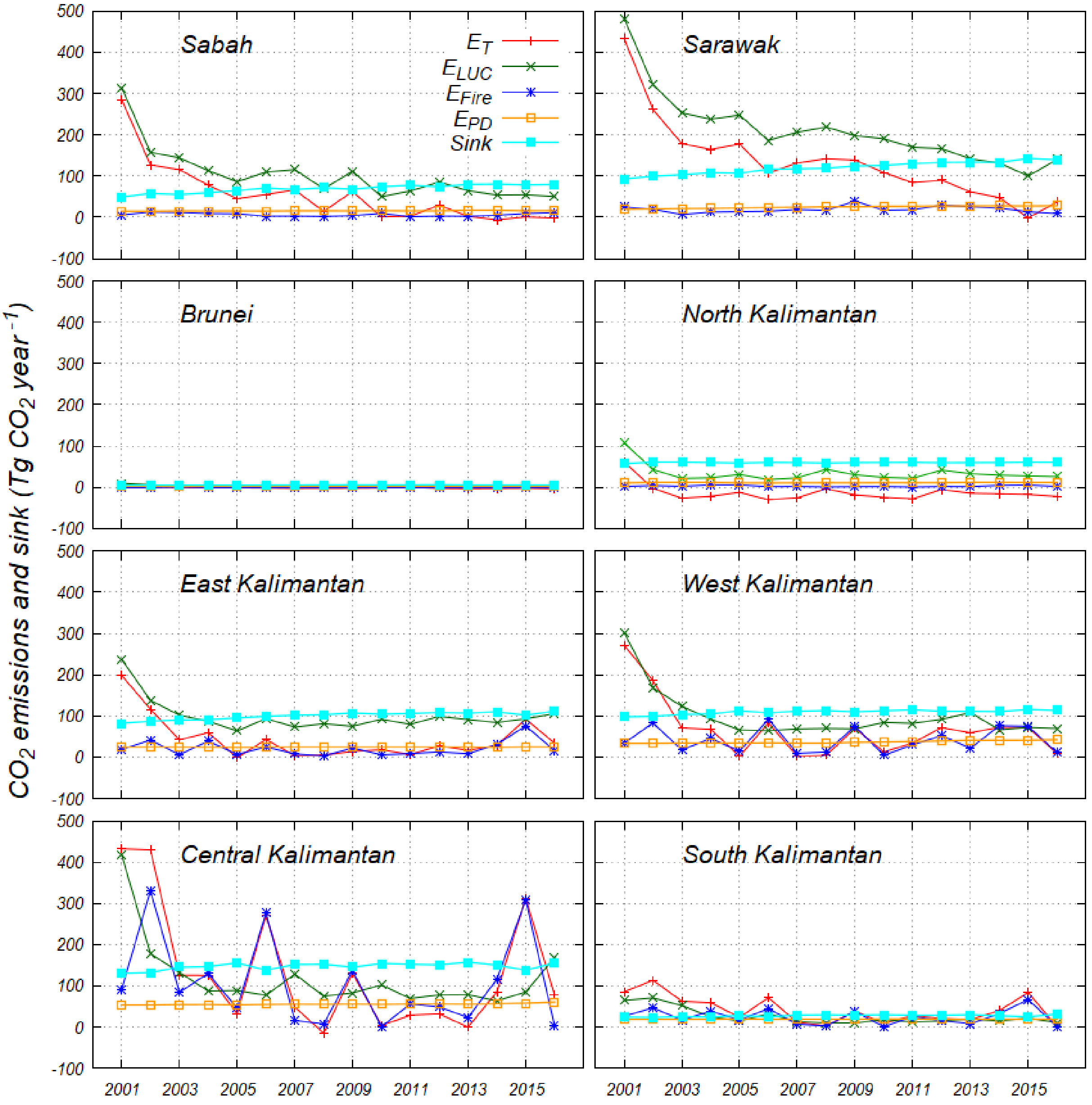

Figure S3. Interannual variations in CO_2 emissions and uptake (Tg CO_2 year^{-1}) in the eight regions from 2001 to 2016. Total net CO_2 emissions (E_T) are in red, emissions from land use change (E_{LUC}) are in green, emissions from forest and peat fires (E_{Fire}) are in blue, emissions from oxidative peat decomposition (E_{PD}) are in orange, and CO_2 uptake (*Sink*) by biomass growth is in light blue. E_T was calculated by subtracting *Sink* from the sum of E_{LUC}, E_{Fire} and E_{PD}.

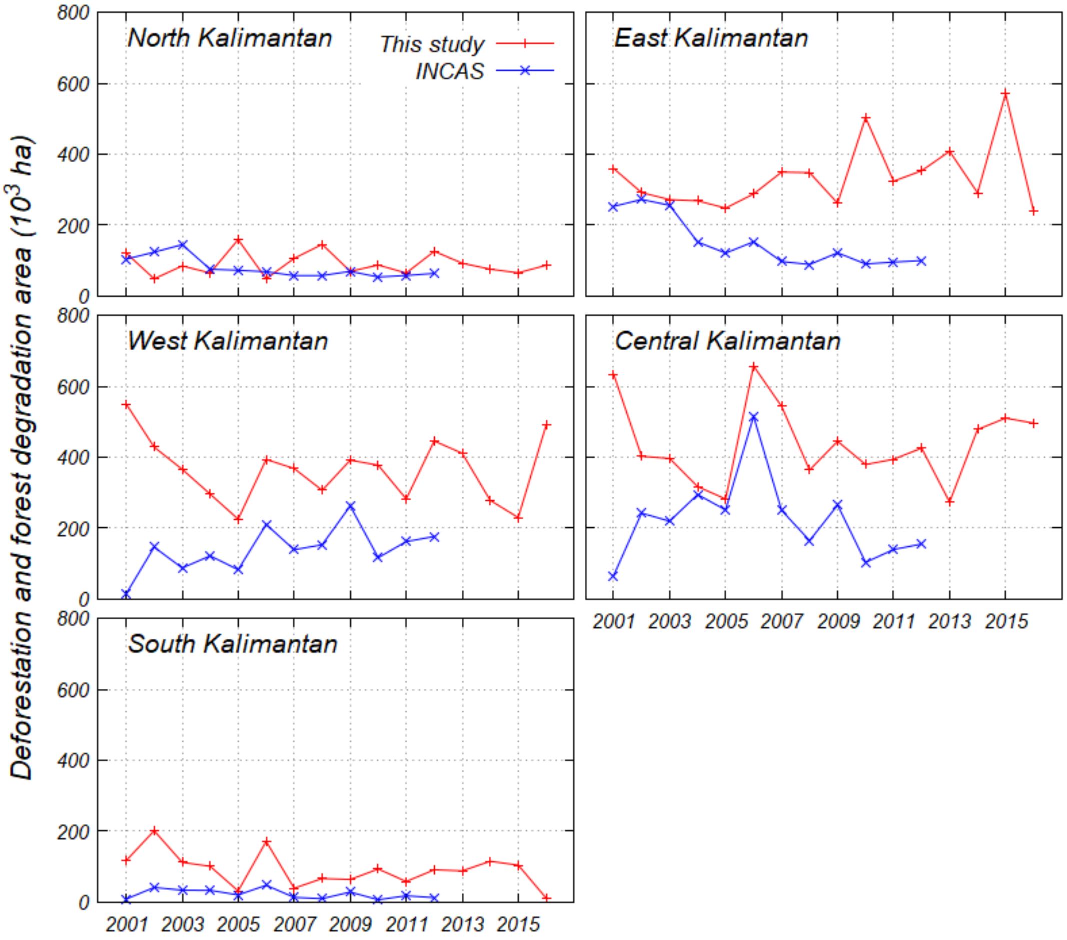

Figure S4. Comparison of interannual variation in the area of land use change in five Indonesian provinces in Borneo from 2001 to 2016. The results of this study (red) show the changed areas from forest to the other land use category. The results of INCAS[1] (blue) show the deforestation and forest degradation areas from 2001 to 2012.

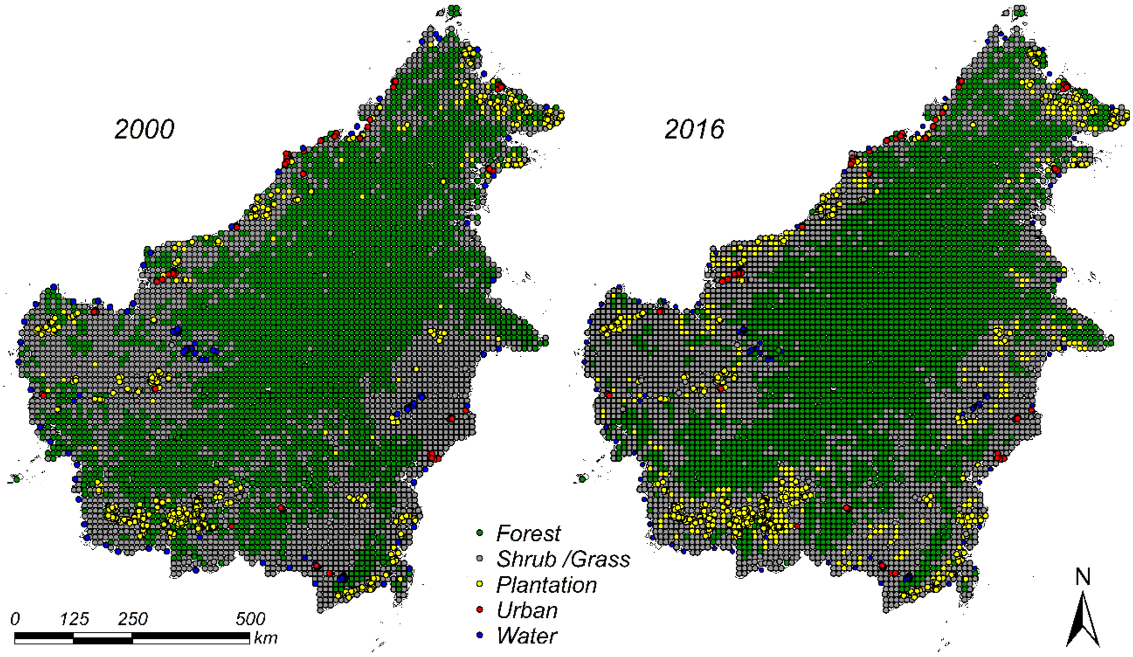

Figure S5. Supervised data from 2000 and 2016. Land use was categorized into five types: forest (green), shrub/grassland (gray), plantation (yellow), urban (red), and water (blue). Maps were created with ArcMap version 10.5 (https://www.arcgis.com/).

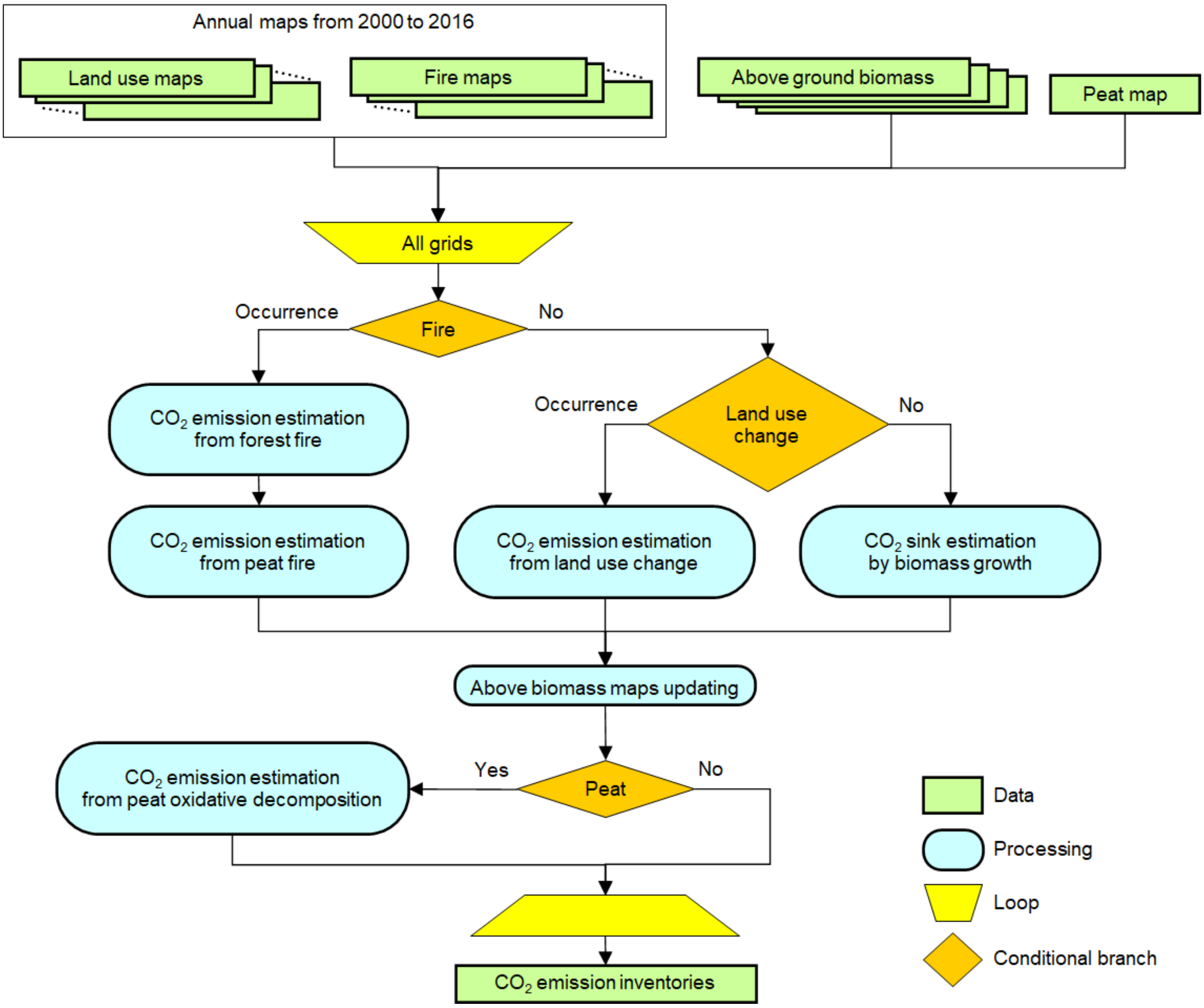

Figure S6. Processing procedure for CO_2 emission estimation from land use change, forest and peat fires, and peat oxidative decomposition.

Table S1. Comparison of deforestation rates (% year^{-1}) between our results and those of previous studies.

Reference	Previous study	This study	Period	Data
Langner et al. (2007)[2]	1.7	0.3	2002–2005	MODIS
Miettinen et al. (2011)[3]	1.3	0.7	2000–2010	MODIS
Bontemps et al. (2012)[4]	1.3–2.7	0.7	2000–2008	SPOT/Vegetation
Hayashi et al. (2015)[5]	2.4	0.5	2004–2007	ICESat/GLAS
Gaveau et al. (2016)[6]	0.7	0.6	2000–2015	Landsat

Table S2. Comparison of annual CO_2 emissions (Tg CO_2 year^{-1}) from land use change in five Indonesian provinces in Borneo from 2001 to 2012.

Region	CO_2 emissions (Tg CO_2 year^{-1})			
	Eavg	Emax	Emin	INCAS[1]
North Kalimantan	25.16	29.87	20.25	24.37
East Kalimantan	57.27	58.17	56.23	46.85
West Kalimantan	70.57	74.87	64.99	49.39
Central Kalimantan	73.20	75.50	70.10	68.91
South Kalimantan	19.64	21.99	17.19	15.73
Total	245.84	260.40	228.76	205.25

Table S3. Comparison of average annual CO_2 emissions (Tg CO_2 year^{-1}) from forest and peat fires from 2000 and 2016 with GFED4.1s.

Region	CO_2 emissions (Tg CO_2 year^{-1})			
	Eavg	Emax	Emin	GFED4.1s
Borneo	217.27	429.98	98.37	177.36
Sabah	6.08	12.08	2.55	2.03
Sarawak	18.69	36.38	8.90	3.89
Brunei	0.22	0.40	0.11	0.11
North Kalimantan	2.42	3.86	1.52	0.87
East Kalimantan	20.36	42.47	7.63	10.40
West Kalimantan	40.68	79.62	17.66	32.44
Central Kalimantan	104.68	204.72	50.06	92.55
South Kalimantan	24.14	50.44	9.94	35.07

Table S4. Comparison of average annual CO_2 emission (Tg CO_2 year^{-1}) from peat fires in five Indonesian provinces in Borneo from 2001 to 2012 with INCAS[1].

Region	CO_2 emissions (Tg CO_2 year^{-1})			
	Eavg	Emax	Emin	INCAS
North Kalimantan	0.82	1.85	0.36	0.02
East Kalimantan	8.85	19.84	3.75	0.57
West Kalimantan	21.21	44.44	10.05	6.41
Central Kalimantan	75.92	161.04	35.39	24.34
South Kalimantan	17.57	38.42	7.66	2.33
Total	124.37	265.59	57.21	33.67

Table S5. Comparison of peat areas in the study area with INCAS[1]. The peat distributions were referenced from Gumbricht et al. (2017)[7].

Region	Entire area (10^6 ha)	Peat area of this study (10^6 ha)	Peat area of INCAS (10^6 ha)
Sabah	7.40	0.68	–
Sarawak	12.38	1.23	–
Brunei	0.58	0.08	–
North Kalimantan	7.01	0.62	–
East Kalimantan	12.57	1.08	0.33
West Kalimantan	14.67	2.12	1.61
Central Kalimantan	15.36	3.25	2.66
South Kalimantan	3.74	0.72	0.11

Table S6. Comparison of the average annual CO_2 emissions (Tg CO_2 $year^{-1}$) from oxidative peat decomposition in five Indonesian provinces in Borneo from 2000 to 2001 with INCAS[1].

Region	CO_2 emissions (Tg CO_2 $year^{-1}$)			
	Eavg	Emax	Emin	INCAS
North Kalimantan	10.13	12.66	7.50	3.70
East Kalimantan	24.09	30.36	17.69	2.45
West Kalimantan	35.29	44.57	26.46	36.92
Central Kalimantan	54.81	69.21	40.33	54.60
South Kalimantan	19.54	25.43	14.24	1.01
Total	143.87	182.23	106.22	98.68

Table S7. Comparison of the average annual CO_2 sink (Tg CO_2 year^{-1}) of forest areas in five Indonesian provinces in Borneo from 2000 to 2012 with INCAS[1]. The CO_2 sink (Tg CO_2 year^{-1}) of the entire area including shrub/grass and plantation areas is shown in the parentheses in the second row.

Region	CO_2 sink (Tg CO_2 year^{-1})			
	Eavg	Emax	Emin	INCAS
North Kalimantan	51.31<br>(59.55)	53.82<br>(62.67)	47.88<br>(55.48)	0.28
East Kalimantan	63.42<br>(97.77)	65.53<br>(100.62)	60.51<br>(93.27)	0.72
West Kalimantan	65.18<br>(108.13)	73.76<br>(117.45)	53.50<br>(94.15)	0.52
Central Kalimantan	95.34<br>(146.18)	107.63<br>(160.25)	78.36<br>(125.45)	0.94
South Kalimantan	7.88<br>(26.97)	8.08<br>(27.84)	7.56<br>(25.60)	0.22
Total	283.13<br>(438.60)	308.82<br>(468.83)	247.81<br>(393.95)	2.68

Table S8. Number of supervised data in each land use category.

Land use category	2000	2001–2015	2016
Forest	3406	2972	3082
Shrub/Grass	2440	2121	2527
Plantation	504	434	742
Urban	166	162	167
Water	152	150	151
Total	6668	5839	6669

Table S9. Index list.

Index	Equation	No.
Ratio Vegetation Index	$\mathrm{RVI} = \frac{band2}{band1}$	1
Difference Vegetation Index	$\mathrm{DVI} = band2 - band1$	2
Normalized Difference Vegetation Index	$\mathrm{NDVI} = \frac{band2 - band1}{band2 + band1}$	3
Soil−Adjusted Vegetation Index	$\mathrm{SAVI} = \frac{1.5\ (band2 - band1)}{(band2 + band1 + 0.5)}$	4
Modified Soil Adjusted Vegetation Index	$\mathrm{MSAVI} = \frac{2\ band2 + 1 - \sqrt{(2\ band2 + 1)^2 - 8(band2 - band1)}}{2}$	5
Normalized Difference Infrared Index 6	$\mathrm{NDII6} = \frac{band2 - band6}{band2 + band6}$	6
Normalized Difference Infrared Index 7	$\mathrm{NDII7} = \frac{band2 - band7}{band2 + band7}$	7
Normalized Difference Water Index	$\mathrm{NDWI} = \frac{band2 - band5}{band2 + band5}$	8
Enhanced Vegetation Index 1	$\mathrm{EVI1} = \frac{2.5\ (band2 - band1)}{(band2 + 6\ band1 - 7.5\ band3 + 1)}$	9
Enhanced Vegetation Index 2	$\mathrm{EVI2} = \frac{2.5\ (band2 - band1)}{(band2 + 2.4\ band1 + 1)}$	10
Green Chromatic Coordinate	$\mathrm{GCC} = \frac{band4}{band1 + band3 + band4}$	11
Generalized Difference Vegetation Index	$\mathrm{GDVI} = \frac{band2 - band4}{band2 + band4}$	12
Visible Atmospherically Resistant Index	$\mathrm{VARI} = \frac{band4 - band1}{band1 + band4 - band3}$	13

Table S10. Time series data list. Day_{Max} and Day_{Min} in Eq. (17) denote the day of the year when the maximum and minimum values were obtained, respectively. D is the number of valid observation data in a year, and V is the valid observation value.

Time series data	Equation	No
Maximum	$Max =$ The maximum value over the year.	14
Minimum	$\mathrm{M}in =$ The minimum value over the year.	15
Range	$\mathrm{Rang} = Max - Min$	16
Trend	$\mathrm{Trend} = \frac{Rang}{Day_{Max} - Day_{Min}}$	17
Average	$\mathrm{Avg} = \frac{1}{D}\sum_{d=1}^{D} V_d$	18
Standard deviation	$\mathrm{Sd} = \sqrt{\frac{1}{D}\sum_{d=1}^{D}(V_i - Avg)^2}$	19
Skewness	$\mathrm{Skew} = \frac{1}{D}\sum_{d=1}^{D}\left(\frac{V_d - Avg}{Sd}\right)^3$	20
Kurtosis	$\mathrm{Kurt} = \frac{1}{D}\sum_{d=1}^{D}\left(\frac{V_d - Avg}{Sd}\right)^4$	21

Table S11. Annual aboveground biomass (AGB) growth (t dry matter ha^{-1} $year^{-1}$) for each land use category. The biomass growth used in this study is the average of the reference data for each land use category. L20 and O20 refer to less or more than 20 years, respectively.

Land use category		Biomass growth	Reference data		
			Biomass growth	Type	Reference
Forest	Non−peat	13.0 (L20)<br>3.4 (O20)	13.0 (L20)<br>3.4 (O20)	Tropical rain forest	IPCC (2006)[8]
	Peat	8.6	10.9	Peat forests (drought)	Miyamoto et al. (2016)[9]
			3.8	Peat forests (post drought)	Miyamoto et al. (2016)[9]
			8.1	Peat forests	Miyamoto et al. (2016)[9]
			11.6	Intact peat swamp forest	Basuki et al. (2018)[10]
Shrub/Grass	Non−peat	2.0 (L20)<br>1.0 (O20)	2.0 (L20)<br>1.0 (O20)	Tropical shrubland	IPCC (2006)[8]
	Peat	7.9	7.9	Logged peat swamp forest	Basuki et al. (2018)[10]
Plantation	Non−peat	3.7	3.7	Oil palm on mineral soils	Khasanah et al. (2015)[11]
	Peat	3.5	3.5	Oil palm on peat	Khasanah et al. (2015)[11]
Urban	–	0.0	0.0	–	–
Water	–	0.0	0.0	–	–

Table S12. Matrix of aboveground biomass (AGB) change between two consecutive years. AGB growth (t dry matter ha^{-1} $year^{-1}$) and AGB associated with land use change for each land use category referenced from Table S11. L20 and O20 refer less and more than 20 years, respectively. The '+' mark refers to adding a value to the previous biomass in the same category, while only numbers without the mark refer to setting a value to AGB for the current year.

Previous year (Before change)		Current year (After change)				
		Forest	Shrub/Grass	Plantation	Urban	Water
Forest	Non−peat	+13.0 (L20)<br>+3.4 (O20)	2.0	3.7	0.0	0.0
	Peat	+8.6	7.9	3.5		
Shrub/Grass	Non−peat	13.0 (L20)<br>3.4 (O20)	+2.0 (L20)<br>+1.0 (O20)	3.7	0.0	0.0
	Peat	8.6	+7.9	3.5		
Plantation	Non−peat	–	–	+3.7	–	–
	Peat			+3.5		
Urban	Non−peat	–	2.0	3.7	0.0	0.0
	Peat		7.9	3.5		
Urban	Non−peat	–	2.0	3.7	0.0	0.0
	Peat		7.9	3.5		

Table S13. Conversion factor (%) for the belowground biomass (BGB) and the sum of wood debris and leaf litter (WDL) from the aboveground biomass (AGB). The conversion factor is the average of the ratio of BGB or WDL to AGB for each set of candidate data in the references. For example, the conversion factor in BGB for Shrub/Grass is {(24.6/180.4)+(16.3/119.6)+(7.0/26.3)}/3=18.0. Furthermore, the conversion factor for plantations was calculated using the breakdown of plantation area according to Gaveau et al. (2016)[6] with the ratio of 85.7% for oil palm and 14.3% for Acacia. The reference data were from Borchard et al. (2019)[12] for No. 1, Krisnawati et al. (2015)[13] for Nos. 2 and 3, Hergoualc'h and Verchot (2011)[14] for Nos. 4, 6, 7, 8, 9, 10 and 11, and Verwer and van der Meer (2010)[15] for No. 5.

Land use category	Conversion factor (%)		Reference data				
	BGB	WDL	No	AGB	BGB	WDL	Description
Forest (Non−peat)	22.3	5.5	1	242.8	54.2	13.4	Secondary forest (≧50 years)
Forest (Peat)	21.5	19.9	2	275.5	69.1	61.7	Primary Swamp Forest
			3	170.5	43.3	49.7	Secondary Swamp Forest
			4	398.9	54.4	28.5	Virgin peat swamp forest
			5	338.8	74.0	70.8	Undisturbed peat swamp forest
Shrub/Grass (Non−peat)	26.6	0.0	6	26.3	7.0	0.0	Crop and Shrub
Shrub/Grass (Peat)	18.0	4.8	7	180.4	24.6	12.9	Logged Peat Forest
			8	119.6	16.3	8.5	Fired Peat Forest
			9	26.3	7.0	0.0	Crop and Shrub
Plantation	24.1	6.8	10	51.3	13.1	2.5	Oil Palm
			11	44.3	7.0	8.1	Acacia
Urban	0	0	–	–	–	–	–
Water	0	0	–	–	–	–	–

Table S14. Burning efficiency (BE: 0 to 1) and emission factor (EF: g CO_2 kg dry matter^{-1}) for each land use category. BE and EF are the average of the references.

Land use category	BE	EF	Reference		
			BE	EF	Reference
Forest	0.12	1643	—	1643	Akagi et al. (2011)[16]
			0.12	—	van der Werf et al. (2017)[17]
Forest (peat)	0.86	1800	—	1710	Huijnen et al. (2016)[18]
			0.86	1889	Wooster et al. (2018)[19]
Shrub/Grass	0.71	1616	—	1710	Akagi et al. (2011)[16]
			0.9	1613	Mieville et al. (2010)[20]
			0.4	1613	Mieville et al. (2010)[20]
			0.9	1613	Mieville et al. (2010)[20]
			0.6	1567	Mieville et al. (2010)[20]
			0.75	1580	Mieville et al. (2010)[20]
Shrub/Grass (Peat)	0.83	1722	—	1594	Huijnen et al. (2016)[18]
			0.772	1564	Stockwell et al. (2016)[21]
			0.81	1775	Wooster et al. (2018)[19]
			0.95	2117	Wooster et al. (2018)[19]
			0.79	1561	Smith et al. (2018)[22]
Plantation	0.60	1515	0.6	1515	Mieville et al. (2010)[20]
Plantation (Peat)	0.84	1677	0.838	1703	Christian et al. (2003)[23]
			0.84	1651	Smith et al. (2018)[22]
Urban	0.0	0	—	—	—
Water	0.0	0	—	—	—

Table S15. Burned peat depth (m) for each land use category. The burned depth represents the average of the results reported.

Land use category	Burned depth	Reference	
		Burned depth	Reference
Forest	0.36	0.51	Page et al. (2002)[24]
		0.33	Ballhorn et al. (2009)[25]
		0.23	Simpson et al. (2016)[26]
Shrub/Grass	0.28	0.23	Simpson et al. (2016)[26]
		0.34	Stockwell et al. (2016)[21]
		0.27	Konecny et al. (2016)[27]
Plantation	0.25	0.23	Simpson et al. (2016)[26]
		0.27	Konecny et al. (2016)[27]
Urban	0	—	—
Water	0	—	—

Table S16. Decreasing rate of burned depth according to the number of repeated fires. The burned depths are based on Konecny et al. (2016)[27].

Repeated fire	Decreasing rate
The first fire	1.0
The second fire	10/17
The third fire	6/17
After the fourth fire	2/17

Table S17. Peat bulk density (BulkD; g cm^{-3}) for each land use category. The bulk densities were determined as the average of the references.

Land use category	Depth (cm)	Bulk density	Reference	
			Bulk density	Reference
Forest	0－10	0.12	0.14	Krisnawati et al. (2021)[28]
			0.13	Krisnawati et al. (2021)[28]
			0.117	Konecny et al. (2016)[27]
			0.11	Itoh et al. (2017)[29]
	10－20	0.16	0.25	Krisnawati et al. (2021)[28]
			0.24	Krisnawati et al. (2021)[28]
			0.117	Konecny et al. (2016)[27]
			0.13	Könönen et al. (2015)[30]
			0.11	Itoh et al. (2017)[29]
			0.09	Itoh et al. (2017)[29]
	20－30	0.19	0.25	Krisnawati et al. (2021)[28]
			0.24	Krisnawati et al. (2021)[28]
			0.09	Itoh et al. (2017)[29]
	30－40	0.20	0.25	Krisnawati et al. (2021)[28]
			0.24	Krisnawati et al. (2021)[28]
			0.10	Itoh et al. (2017)[29]
Shrub/Grass	0－10	0.18	0.18	Krisnawati et al. (2021)[28]
			0.18	Krisnawati et al. (2021)[28]
			0.115	Konecny et al. (2016)[27]
			0.24	Itoh et al. (2017)[29]
	10－20	0.18	0.22	Krisnawati et al. (2021)[28]
			0.21	Krisnawati et al. (2021)[28]
			0.115	Konecny et al. (2016)[27]
			0.20	Könönen et al. (2015)[30]
			0.14	Itoh et al. (2017)[29]
	20－30	0.19	0.22	Krisnawati et al. (2021)[28]
			0.21	Krisnawati et al. (2021)[28]
			0.14	Itoh et al. (2017)[29]
	30－40	0.19	0.22	Krisnawati et al. (2021)[28]
			0.21	Krisnawati et al. (2021)[28]
			0.14	Itoh et al. (2017)[29]

Plantation	0－10	0.16	0.12	Husnain et al. (2014)[31]
			0.15	Husnain et al. (2014)[31]
			0.21	Husnain et al. (2014)[31]
			0.24	Itoh et al. (2017)[29]
			0.20	Itoh et al. (2017)[29]
			0.089	Hooijer et al. (2012)[32]
			0.087	Hooijer et al. (2012)[32]
	10－20	0.15	0.18	Könönen et al. (2015)[30]
			0.12	Husnain et al. (2014)[31]
			0.15	Husnain et al. (2014)[31]
			0.21	Husnain et al. (2014)[31]
			0.23	Itoh et al. (2017)[29]
			0.089	Hooijer et al. (2012)[32]
			0.087	Hooijer et al. (2012)[32]
	20－30	0.14	0.23	Itoh et al. (2017)[29]
			0.089	Hooijer et al. (2012)[32]
			0.087	Hooijer et al. (2012)[32]
Urban	—	—	—	—
Water	—	—	—	—

Table S18. Emission factor (t CO_2–C ha^{-1} year^{-1}) for peat oxidative decomposition. The emission factor represents the average of multiple candidates for the land use category (IPCC 2014[33]).

Land use category		Emission factor	Reference (IPCC 2014[33])	
			Emission factor	Land use category
Forest	Not drained	0.0	0.0	Peat swamp forest
	Drained	5.3	5.3	Forest Land and cleared Forest Land (shrubland), drained
Shrub/Grass		7.45	5.3	Forest Land and cleared Forest Land (shrubland), drained
			9.6	Grassland, drained
Plantation		15.3	15.0	Plantations, drained, unknown or long rotations
			20.0	Plantations, drained, short rotations, e.g. acacia
			11.0	Plantations, drained, oil palm
Urban		7.45	5.3	Forest Land and cleared Forest Land (shrubland), drained
			9.6	Grassland, drained
Water		0.0	0.0	Water

References

1. Indonesian National Carbon Accounting System (INCAS). Available at: http://incas.menlhk.go.id/ (Accessed: 23th December 2021)
2. Langner, A., Miettinen, J., & Siegert, F. Land cover change 2002–2005 in Borneo and the role of fire derived from Modis imagery. *Global Change Biology* **13,** 2329–2340 (2007).
3. Miettinen, J., Shi, C. & Liew, S. C. Deforestation rates in Insular Southeast Asia between 2000 and 2010. *Global Change Biology* **17,** 2261–2270 (2011).
4. Bontemps, S., Langner, A. & Defourny, P. Monitoring Forest changes in Borneo on a yearly basis by an object-based change detection algorithm using spot-vegetation time series. *International Journal of Remote Sensing* **33,** 4673–4699 (2012).
5. Hayashi, M., Saigusa, N., Yamagata, Y. & Hirano, T. Regional forest biomass estimation using icesat/glas spaceborne lidar over Borneo. *Carbon Management* **6,** 19–33 (2015).
6. Gaveau, D. L. *et al.* Rapid conversions and avoided deforestation: Examining four decades of industrial plantation expansion in Borneo. *Scientific Reports* **6,** (2016).
7. Gumbricht, T. *et al.* Tropical and subtropical wetlands distribution version 2. *Tropical and Subtropical Wetlands Distribution version 2* (2017). doi:10.17528/cifor/data.00058
8. Intergovernmental Panel on Climate Change (IPCC). *2006 IPCC Guidelines for National Greenhouse Gas Inventories*. Hayama, Japan: Institute for Global Environmental Strategies (IGES). (2006).
9. Miyamoto, K. *et al*. Chapter 10: Forest Structure and Productivity of Tropical Heath and Peatland Forests. In M. Osaki & N. Tsuji (Eds.), *Tropical Peatland Ecosystems,* 151–166. Springer. (2016).
10. Basuki, I., Kauffman, J. B., Peterson, J., Anshari, G. & Murdiyarso, D. Land cover changes reduce net primary production in tropical coastal peatlands of West Kalimantan, Indonesia. *Mitigation and Adaptation Strategies for Global Change* **24,** 557–573 (2018).
11. Khasanah, N., van Noordwijk, M. & Ningsih, H. Aboveground carbon stocks in oil palm plantations and the threshold for carbon-neutral vegetation conversion on mineral soils. *Cogent Environmental Science* **1,** 1119964 (2015).
12. Borchard, N. *et al.* Deep soil carbon storage in tree-dominated land use systems in tropical lowlands of Kalimantan. *Geoderma* **354,** 113864 (2019).
13. Krisnawati, H. *et al*. Standard Methods for Estimating Greenhouse Gas Emissions from Forests and Peatlands in Indonesia (Version 2). Research, Development and Innovation Agency of the Ministry of Environment and Forestry. Bogor, Indonesia. (2015).
14. Hergoualc'h, K. & Verchot, L. V. Stocks and fluxes of carbon associated with land use change in Southeast Asian tropical peatlands: A Review. *Global Biogeochemical Cycles* **25,** (2011).
15. Verwer, C. C. & van der Meer, P. J. Carbon pools in tropical peat forest: towards a reference value for forest biomass carbon in relatively undisturbed peat swamp forests in Southeast Asia. *Alterra-report 2108*. Wageningen, Alterra. (2010).
16. Akagi, S. K. *et al.* Emission factors for open and domestic biomass burning for use in atmospheric models. *Atmospheric Chemistry and Physics* **11,** 4039–4072 (2011).
17. van der Werf, G. R. *et al.* Global Fire Emissions Estimates during 1997–2016. *Earth System Science Data* **9,** 697–720 (2017).
18. Huijnen, V. *et al.* Fire carbon emissions over Maritime Southeast Asia in 2015 largest since 1997. *Scientific Reports* **6,** (2016).
19. Wooster, M. *et al.* New tropical peatland gas and Particulate Emissions Factors indicate 2015 Indonesian fires released far more particulate matter (but less methane) than current inventories imply. *Remote Sensing* **10,** 495 (2018).
20. Mieville, A. *et al.* Emissions of gases and particles from biomass burning during the 20th century using satellite data and an historical reconstruction. *Atmospheric Environment* **44,** 1469–1477 (2010).
21. Stockwell, C. E. *et al.* Field measurements of trace gases and aerosols emitted by peat fires incentral Kalimantan, Indonesia, during the 2015 el niño. *Atmospheric Chemistry and Physics* **16,** 11711–11732 (2016).
22. Smith, T. E., Evers, S., Yule, C. M. & Gan, J. Y. In situ tropical peatland fire emission factors and their variability, as determined by field measurements in Peninsula Malaysia. *Global Biogeochemical Cycles* **32,** 18–31 (2018).
23. Christian, T. J. Comprehensive laboratory measurements of biomass-burning emissions: 1. emissions from Indonesian, African, and other fuels. *Journal of Geophysical Research* **108,** (2003).

24. Page, S. E. *et al.* The amount of carbon released from peat and forest fires in Indonesia during 1997. *Nature* **420,** 61–65 (2002).
25. Ballhorn, U., Siegert, F., Mason, M. & Limin, S. Derivation of burn scar depths and estimation of carbon emissions with Lidar in Indonesian peatlands. *Proceedings of the National Academy of Sciences* **106,** 21213–21218 (2009).
26. Simpson, J. *et al.* Tropical peatland burn depth and combustion heterogeneity assessed using UAV photogrammetry and Airborne Lidar. *Remote Sensing* **8,** 1000 (2016).
27. Konecny, K. *et al.* Variable carbon losses from recurrent fires in drained tropical peatlands. *Global Change Biology* **22,** 1469–1480 (2016).
28. Krisnawati, H. *et al.* Carbon balance of tropical peat forests at different fire history and implications for carbon emissions. *Science of The Total Environment* **779,** 146365 (2021).
29. Itoh, M., Okimoto, Y., Hirano, T. & Kusin, K. Factors affecting oxidative peat decomposition due to land use in tropical peat swamp forests in Indonesia. *Science of The Total Environment* **609,** 906–915 (2017).
30. Könönen, M. *et al*. Physical and chemical properties of tropical peat under stabilised land uses. *Mires and Peat* **16**, 1–13 (2015).
31. Husnain, H. *et al.* CO2 emissions from tropical drained peat in Sumatra, Indonesia. *Mitigation and Adaptation Strategies for Global Change* **19,** 845–862 (2014).
32. Hooijer, A. *et al.* Subsidence and carbon loss in drained tropical peatlands. *Biogeosciences* **9,** 1053–1071 (2012).
33. Intergovernmental Panel on Climate Change (IPCC). *2013 Supplement to the 2006 IPCC Guidelines for National Greenhouse Gas Inventories: Wetlands*. (2014).
